# The impact and feasibility of a brief, virtual, educational intervention for home healthcare professionals on Parkinson’s Disease and Related Disorders: pilot study of I SEE PD Home

**DOI:** 10.1186/s12909-022-03430-7

**Published:** 2022-06-28

**Authors:** Serena P. Hess, Melissa Levin, Faizan Akram, Katheryn Woo, Lauren Andersen, Kristie Trenkle, Patricia Brown, Bichun Ouyang, Jori E. Fleisher

**Affiliations:** 1 Department of Neurological Sciences, Section of Movement Disorders, Rush University Medical Center, 1725 W. Harrison Street, Suite 755, Chicago, IL 60612 USA; 2 Chicago Medical School – Rosalind Franklin University, 3333 Green Bay Road, North Chicago, IL 60064 USA; 3 Loyola University Chicago College of Arts and Sciences, 1032 W. Sheridan Road, Chicago, IL 60660 USA; 4 Department of Physical Therapy, Rush University Medical Center, 1725 W. Harrison Street, Suite 440, Chicago, IL 60612 USA; 5 Rush Physical Therapy, Select Medical, 1725 W. Harrison Street, Chicago, IL 60612 USA; 6 Department of Occupational Therapy, Rush University Medical Center, 1725 W. Harrison Street, Suite 440, Chicago, IL 60612 USA; 7 Memorycare Corporation, 634 Brooklyn Drive, Aurora, IL 60502 USA

**Keywords:** Interprofessional education, Allied health, Home healthcare, Parkinson’s Disease, Parkinsonism, Virtual reality, Physical therapy, Occupational therapy, Speech-language pathology, Neurodegenerative

## Abstract

**Background:**

Individuals with advanced Parkinson’s Disease (PD) and Parkinson-related disorders (PRD) are frequently referred for home allied therapies and nursing care, yet home healthcare professionals have limited training in PD/PRD. While recognizing the need for such care, patients and families report home healthcare professionals are unfamiliar with these conditions, which may be driven by neurophobia and may contribute to suboptimal care and early termination of services. We sought to determine the feasibility and effects of a virtual, multimodal educational intervention on PD knowledge, confidence, and empathy among home health professionals.

**Methods:**

Home health nurses, occupational therapists, physical therapists and physical therapy assistants, and speech-language pathologists participated in a daylong, virtual symposium on advanced PD/PRD, combining focused lectures, discipline-specific breakout sessions, immersive virtual reality vignettes, and interactive panels with both patients and families, and movement disorders and home healthcare experts. Participants completed online pre- and post-symposium surveys including: demographics; PD/PRD knowledge (0–10 points possible); empathy (Interpersonal Reactivity Index); and 10-point scales of confidence with and attitudes towards individuals with PD/PRD, respectively. Pre-post intervention changes and effect sizes were evaluated with paired t-tests and Cohen’s d. We performed qualitative analyses of post-symposium free-text feedback using a grounded theory approach to identify participants’ intentions to change their practice.

**Results:**

Participants had a mean improvement of 3.1 points on the PD/PRD knowledge test (*p* < 0.001, d = 1.97), and improvement in confidence managing individuals with PD/PRD (*p* = 0.0003, d = .36), and no change in empathy. The interactive, virtual format was rated as effective by 95%. Common themes regarding symposium-motivated practice change included: interdisciplinary collaboration; greater involvement and weighting of the patient and caregiver voice in care plans; attention to visit scheduling in relation to patient function; recognition and practical management of the causes of sudden change in PD/PRD, including infections and orthostatic hypotension.

**Conclusions:**

A virtual, multimodal, brief educational pilot intervention improved PD/PRD-specific knowledge and confidence among home healthcare nurses and allied health professionals. Future studies are necessary to test the short- and long-term effects of this intervention more broadly and to investigate the impact of this education on patient and caregiver outcomes.

**Supplementary Information:**

The online version contains supplementary material available at 10.1186/s12909-022-03430-7.

## Introduction

Parkinson’s Disease (PD) is the second most common neurodegenerative disease worldwide, with prevalence expected to double by 2060 [1, 2]. As PD progresses, patients have increased dependence and healthcare utilization, including both outpatient, acute or inpatient, and home care [3, 4]. Several interdisciplinary, PD-focused, educational interventions for healthcare professionals have demonstrated improvements in participants’ knowledges, attitudes, and practices in the US [5, 6], Netherlands [7], and Cameroon [8]. However, these interventions have largely attracted providers with a dedicated PD focus in their outpatient practice setting. [6, 7].

In 2015, 3.5 million US Medicare beneficiaries received 119 million home healthcare visits, of which 36% were skilled rehabilitation, including physical, occupational, or speech therapy [9] Literature supports the benefits of home-based rehabilitation on decreasing fall risk in homebound older adults [10] and improving functional recovery after hip fracture [11], and the benefits of home-based nursing in preventing rehospitalizations and improving patient outcomes [12]. To our knowledge, however, there are no interventions specifically focused on educating home nursing and rehabilitation professionals about advanced PD and related disorders such as Lewy Body Dementia (LBD), Progressive Supranuclear Palsy (PSP), Multiple System Atrophy (MSA), and Corticobasal Syndrome (CBS), hereafter referred to as Parkinson-related disorders (PRD). This is particularly important for multiple reasons: first, these healthcare professionals are the most likely to see these individuals as they become homebound, second, this population is at high risk of falls, hip fractures, hospitalization, and institutionalization, and third, as described above, home health interventions have demonstrated benefits in preventing or recovering from these outcomes in other older adult populations [13–15].

In pilot work conducted in New York and replicated in Chicago, our team comprised of a movement disorders specialist, nurse, and social worker, conducted over 500 interdisciplinary home visits with homebound individuals with advanced PD and PRD. Over 91% of visits culminated with allied health or specialty referrals [16–18]. It was not uncommon for participants to report that therapists came to the home once or twice, admitted a lack of PD or PRD knowledge, and promptly discharged the patient despite clear indications for such care. Other participants with prominent dysautonomia were inappropriately referred to the emergency department by home health professionals for asymptomatic hyper- or hypotension [17]. Despite the volume of referrals, no prior literature exists on patient or care partner satisfaction with home healthcare in PD and PRD, or the degree to which knowledge, confidence, and attitudes of the home healthcare professional effects the patient and care partner’s perception of benefit.

Beyond home visits, the lack of PD- and PRD-specific knowledge and training among nurses and allied health professionals has been identified in other contexts. The Parkinson's Disease Medication Protocol Program showed that without targeted, PD education modules, nurses may lack sufficient clinical expertise to effectively manage PD medications, specifically leading to detrimental dopaminergic medication errors and omissions [19]. More broadly, “neurophobia”, or a “fear of neural sciences and clinical neurology” [20] has long been recognized as a barrier to medical students and non-neurologist physicians, as well as dental students, occupational therapy (OT) students, and speech-language pathology (SLP) students [21, 22], with the high perceived level of difficulty of neuroanatomy inversely proportional to levels of interest in learning, understanding, and treating individuals with neurologic disorders across interdisciplinary students. To our knowledge, there have been no specific investigations of neurophobia in home healthcare professionals.

A dire need for PD- and PRD-specific home nurse and allied health professional education was identified, prompting the development of an Interactive Symposium and Educational Experience on PD for Home Health Professionals (I SEE PD Home). We aimed to determine the effect of a multimodal, interactive, virtual educational intervention on PD and PRD knowledge, confidence, and empathy among home healthcare professionals. We hypothesized that an intervention incorporating both content knowledge and experiential learning (i.e., immersive virtual reality modules) could improve knowledge of and attitudes towards individuals living with PD and PRD among home healthcare professionals [23].

## Methods

### Study design and setting

Here, we present a single-center, pre-post intervention study of a daylong, virtual educational symposium. Supported by a Parkinson’s Foundation (PF) Centers of Excellence Community Outreach, Research, and Engagement (CORE) grant, we partnered with our continuing education (CE) office to insure content was robust and eligible for the provision of CE credits for allied health professionals completing the full symposium. Given restrictions due to the SARS-CoV-2 pandemic, this daylong virtual symposium was broadcast via Zoom® from Rush University Medical Center in Chicago, Illinois.

### Participants and recruitment

We designed the symposium specifically for licensed allied home health professionals in the following disciplines, each of whom were eligible for CE credits: registered nurse (RN), occupational therapy (OT), physical therapy (PT), physical therapy assistant (PTA), or speech-language pathology (SLP). Nursing students were invited to participate, but not eligible for CE credits. All participants met these criteria: primarily English-speaking, agreed to attend the entire day of the symposium, and had a working computer, internet connection, and email address.

Our recruitment target was 50 participants based on funding limitations, recruited from nine home healthcare agencies (HHAs). HHAs were invited to participate if the Rush Movement Disorders Clinic had referred individual patients to the agency within the past year, without significant negative feedback or concerns about the agency from referring providers. The study team contacted clinical leaders within the HHAs and asked the leaders to circulate the informational flyer to their internal teams and support participation of healthcare professionals in their respective organizations.

### Intervention

This educational intervention included four components, all focused on PD and PRD: lectures, discipline-specific breakout sessions, a patient and caregiver panel, and an immersive, virtual reality (VR) experience (Table 1). The symposium began with traditional lectures to introduce PD and PRD, including LBD, PSP, MSA, and CBS. The lectures focused on evidence-based management, including in-depth coverage of advanced motor and non-motor symptoms in later stages of PD and PRD. A second lecture focused on urgent situations detected during home healthcare visits, including falls, mental status changes, or concerns about neglect or abuse, with provision and review of critical resources in the PF Aware in Care Kit [24].

**Table 1:** I SEE PD Home agenda and curriculum

Duration	Topic
**30 min**	I SEE PD Home Welcome and Pre-Intervention Surveys
**80 min**	Pearls of Parkinson’s Disease and Related Disorders: What You Need to Know
**30 min**	Virtual Reality Experience Part 1
**60 min**	Discipline-specific breakout sessions
	Life with PD: What Nurses Need to Know
	Life with PD: Overview for Physical and Occupational Therapists
	Life with PD: Overview for Speech-Language Pathologists
**30 min**	Virtual Reality Experience Part 2
**15 min**	Morning Summary
**60 min**	Voice of the Patient & Caregiver – Interactive Panel
**15 min**	Virtual Reality Experience Part 3
**45 min**	Detecting Urgent Situations in the Home: What You Need to Look For
**45 min**	Allied Health Panel with Live Question & Answer Session
**45 min**	Summary, Post-Intervention Surveys, and Evaluation

*I SEE PD Home* Interactive Symposium and Educational Experience on Parkinson’s Disease for Home Health Professionals, *PD* Parkinson’s Disease

Attendees chose to attend one of three breakout sessions: 1) What Nurses Need to Know: covering the importance of medication timing and reconciliation, orthostatic hypotension, and constipation; 2) Overview for Physical and Occupational Therapists: covering fall prevention, home safety assessments, healthy exercise and movement; or 3) Overview for Speech-Language Pathologists: targeting hypophonia, dysarthria, dysphagia, and related issues, assistive devices, and other treatment options. All breakout sessions introduced practical strategies to promote healthy aging-in-place in PD/PRD and were led by a home health professional with PD/PRD expertise in each respective discipline.

After the breakout sessions, the participants were joined on Zoom by a panel of several caregivers and people living with advanced PD/PRD. Panelists introduced themselves, their experience with home health, and illustrated opportunities for benefit, change, and potential impact. Following introductions, symposium participants were able to pose questions to the panelists. Pertinent questions asked by the HHA professional participants of the patients and caregivers included: whether families felt they had sufficient education on PD/PRD when first diagnosed; cues, exercises, and devices that promoted safe aging-in-place in their own homes, and whether they had discovered these on their own or they had been recommended by healthcare professionals; communication tips for people living with PD/PRD; recommendations for scheduling home therapy sessions around motor fluctuations and non-motor symptoms such as daytime sleepiness and apathy.

Participants also completed three 10–12-min sessions of the Dima Lab (Embodied Labs, Los Angeles, CA) immersive VR experience throughout the day. While connected to the symposium’s videoconferencing software, the VR experience was livestreamed such that participants were viewing it in real-time. In the Dima Lab, the viewer inhabits the body of a Lebanese-American woman named Dima with LBD. Across the three vignettes, the viewer, as Dima, interacts with family members in her own home, and with healthcare providers in both outpatient and institutional settings, demonstrating multiple motor and non-motor symptoms along with Dima’s internal monologue. The learning objectives for the Dima Lab included: recognizing symptoms of LBD; helping people with LBD experiencing anxiety, agitation, or hallucinations; and recognizing when emotional or physical burnout, financial burden, or safety may necessitate help from professional care agencies or residential care communities.

The symposium concluded with a question and answer panel with the breakout session leaders and the movement disorders specialist, a final set of summary slides, and the online post-intervention surveys and evaluations. All symposium slides and materials are available in Additional file 2: Appendix B, and annotated facilitator guides for both the patient and caregiver panel and the final expert question and answer panel are provided in Additional file 3: Appendix C.

### Measures

#### Survey structure

Participants completed all demographic, knowledge, and attitude measures in real-time at the beginning of the symposium (pre-intervention) and again at the end of the day (post-intervention) via a link to a secure, electronic database shared by the study team [25]. At both time points, participants were prompted to select the first initial of their first name, color of their first car, and manually enter their childhood home street name, generating a unique identifier to facilitate linkage of pre- and post-intervention assessments. To obtain CE credits, participants completed a standardized CE questionnaire via an online link emailed to them directly from the CE office.

#### Demographic information and experience

Sociodemographic information included: age, gender identity, race, and ethnicity. Participants reported: discipline (registered nurse, physical therapist, physical therapy assistant, occupational therapist, or speech-language pathologist); years since terminal degree; years of experience in home health care specifically; volume of individuals treated with PD/PRD in the past year (categorized as 0, 1–5, 6–10, 11–15, 16–20, or > 20), and whether the participant had any personal or family experience with PD/PRD.

#### PD/PRD knowledge

For this symposium, the study team developed a 10-item, multiple choice survey covering practical knowledge related to the management of individuals with PD/PRD that might reasonably be encountered by and pertinent to home healthcare professionals (Additional file 1: Appendix A). Each item was designed by a member of the study team with survey development experience [26] (JEF) based on the lecture content and following guidance on survey development in medical education, using positive language, avoiding reverse-scored items, providing an appropriate number of and appropriately labeled response options in a single column, and asking the more important items earlier in the survey [27]. A panel of independent movement disorders experts then separately reviewed and revised the prompts and response, followed by an additional round of revisions and review for any items of remaining concern, using a modified Delphi approach [28]. We hypothesized that post-intervention, participants would demonstrate a mean 10% improvement compared to their pre-intervention PD/PRD Knowledge score.

#### Secondary Outcomes: Empathy, confidence, attitudes, and feasibility

We assessed empathy using the Interpersonal Reactivity Index (IRI), a 28-item validated tool to measure change in empathy among nursing students and other healthcare professionals following educational interventions [29]. The IRI is scored as four domain subscales, namely, Perspective Taking, Fantasy, Empathic Concern, and Personal Distress. Each domain is made up of seven items scored on a 5-point Likert scale where higher scores indicate higher empathy. To evaluate the efficacy of the Dima Lab VR PD scenarios, we administered four proprietary questions designed by the VR manufacturer (Embodied Labs) assessing change in confidence and attitudes towards people with PD/PRD [30], where the response options for each prompt were a ten-point Likert scale ranging from strongly disagree [1] to strongly agree [10]. We assessed the feasibility of the I SEE PD Home intervention by tracking attendance in real time and awarding CE credits only once the participant had completed the post-intervention evaluation. We solicited quantitative feedback on the symposium via a standardized CE evaluation, and qualitative feedback from the participants throughout the intervention via live comments (verbal and chat) and open-ended questions in the CE evaluation.

### Data analysis

All pre- and post-intervention data were entered directly into a HIPAA-compliant, secure electronic database by the participants [25]. Data was exported, cleaned, and analyzed in Stata 15 (StataCorp, 2015). Descriptive analysis of the demographics, professional experience, and baseline assessment scores included means/standard deviation or median/interquartile range, as appropriate. We used paired t-tests to assess for change in the pre- and post-intervention PD/PRD Knowledge Test (primary outcome) and in the IRI and VR questionnaires (secondary outcomes), with Cohen’s *d* to calculate effect size, where 0.2, 0.5, and 0.8 signified small, medium, and large effect sizes, respectively [31]. For qualitative feedback, a grounded theory approach was used to code participant free-text survey responses. Two study team members (SH, JEF) independently coded responses, met to discuss emerging themes, and reviewed coded responses in relation to the themes, highlighting illustrative quotations for each theme [32, 33]. As this was a pilot study, our target recruitment goal of 50 participants was based on feasibility and funding restrictions.

## Results

### Participant characteristics

A total of 52 attendees completed the full-day, I SEE PD Home virtual symposium on March 5, 2021. Of these 52 attendees, five nursing students were excluded from study participation as they were ineligible for CE credits. Forty-two of the remaining 47 participants completed the demographics information and 35 participants completed all pre- and post-intervention surveys (Table 2, middle and right columns, respectively); there were no significant differences between the groups. Most attendees were female (90.5%) and between the ages of 35–44 (26.2%) or 45–54 (38.1%). PTs represented 38% of the attendees, followed by: OTs (21.4%), RNs (16.7%), SLPs (7.1%), PTAs (4.8%), and 11.9% “other” or did not disclose their discipline.

**Table 2:** Participant demographics, discipline, and experience with Parkinson’s Disease and related disorders

	Participants completing pre-symposium survey only *N* = 42 % (n)	Participants completing pre- and post-symposium surveys *N* = 35 % (n)
**Age in years,** % (n)		
18–24	4.76 (2)	2.86 (1)
25–34	19.05 (8)	20.00 (7)
35–44	26.19 (11)	25.71 (9)
45–54	38.10 (16)	40.00 (14)
55–64	9.52 (4)	11.43 (4)
75 or older	2.38 (1)	0 (0)
**Female,** % (n)	90.48 (38)	88.57 (31)
**Race,** % (n)		
Caucasian	66.67 (28)	71.43 (25)
African American	16.67 (7)	14.29 (5)
Asian	11.90 (5)	11.43 (4)
Native Hawaiian	2.38 (1)	0 (0)
More than one race	2.38 (1)	2.86 (1)
**Hispanic or Latinx,** % (n)	2.38 (1)	2.86 (1)
**Discipline,** % (n)		
RN	16.67 (7)	17.14 (6)
PT	38.10 (16)	40.00 (14)
PTA	4.76 (2)	5.71 (2)
OT	21.43 (9)	22.86 (8)
SLP	7.14 (3)	8.57 (3)
Other	11.90 (5)	5.71 (2)
**Years since terminal degree,** mean (SD)	16.26 (10.51)	15.97 (9.44)
**Years of experience in home health care,** mean (SD)	7.12 (6.65)	7.26 (6.47)
**In the last year, how many people have you treated with Parkinson's Disease or Lewy Body Dementia?**		
0	23.81 (10)	22.86 (8)
1–5	23.81 (10)	22.86 (8)
6–10	21.43 (9)	17.14 (6)
11–15	14.29 (6)	17.14 (6)
16–20	4.76 (2)	5.71 (2)
21 or more	11.90 (5)	14.29 (5)
**Personal or family experience with Parkinson's Disease or Lewy Body Dementia**, % (n)	26.19 (11)	25.71 (9)

The attendees had a mean of 16.3 years (standard deviation (SD) 10.5) in practice since training, and 7.1 years (SD 6.7) of home health care experience specifically. Nearly one quarter of participants (23.8%) had not treated any patients with PD/PRD in the past year, and another 23.8% reported treating 1–5 such individuals, combined. Eleven participants (26.2%) endorsed personal or family experience with PD/PRD. Participants cared for patients across six states: Illinois, Indiana, Kentucky, Pennsylvania, Texas, and Wisconsin.

### Primary and secondary outcomes

Complete pre- and post-intervention primary outcome data (PD/PRD Knowledge score) was available for 35 individuals, and complete pre- and post-intervention secondary outcome data for 37 (IRI) and 36 (VR questionnaire), respectively. Among the 35 participants completing the PD/PRD Knowledge test pre- and post-intervention, 91.42% (*n* = 32) improved their post-intervention score by our a priori cut-off of at least 10% and the mean improvement was 31.14% (SD 17.62%). As shown in Table 3, home health professionals scored a mean of 4.06 out of 10.0 possible points on the PD/PRD Knowledge test at baseline pre-intervention (SD 1.53), and showed a mean 3.1-point improvement post-intervention (mean 7.17, SD 1.62, *p* < 0.0001), with a large effect size of 1.97.

**Table 3:** Change in knowledge and attitudes pre- and post-intervention

	Pre-intervention Mean (SD)	Post-intervention Mean (SD)	*p*-value	Cohen’s d
**PD/PRD knowledge test total score**, mean (SD)^a^	4.06 (1.53)	7.17 (1.62)	** < 0.0001**	**1.97**
**Interpersonal Reactivity Index,** subscale mean (SD)^b^				
Perspective-Taking	21.19 (3.88)	21.62 (3.83)	0.23	0.11
Fantasy Scale	14.46 (5.52)	13.78 (5.96)	0.16	0.12
Empathic Concern	22.89 (3.88)	23.14 (3.80)	0.58	0.07
Personal Distress	9.68 (5.52)	9.41 (5.62)	0.57	0.05
VR Questionnaire—confidence and attitudes*, median (IQR)^c^				
A person living with Lewy body dementia and/or Parkinson’s disease is able to spend their time meaningfully and can contribute to family and community life	8.50 (3.00)	9.50 (2.00)	**0.01**	0.39
I am confident in my ability to help a person expressing agitation, stress, combativeness, hallucination, or sensory overload as a result of their progressing dementia and/or Parkinson’s disease	7.00 (3.00)	8.00 (2.50)	**0.0003**	0.36
I do or would feel comfortable using an aide from a home health care agency to help care for my loved one living with Lewy Body dementia or Parkinson’s disease	8.50 (5.00)	9.00 (3.00)	0.23	0.12
I feel confident that I am able to positively affect the quality of life of a resident, client, or loved one experiencing later stage Lewy Body dementia or Parkinson’s disease	8.00 (3.00)	8.00 (2.00)	0.12	0.0

^a^ *n* = 35; ^b^ *n* = 37; ^c^ *n* = 36

^*^Questions provided by developers of virtual reality modules

**Bolded** indicates statistical significance, *p* < 0.05, or Cohen’s d consistent with large effect size

We found no change in any of the four IRI empathy domains (*p* = 0.16–0.58, Cohen’s d = 0.05–0.12). In the VR questionnaire, there was a 1-point improvement (Cohen’s d = 0.39) in response to whether participants felt that “a person living with Lewy body dementia and/or Parkinson’s disease is able to spend their time meaningfully and can contribute to family and community life” (pre-intervention mean 8.50 (SD 3.00) vs. post-intervention 9.50 (SD 2.00), *p* = 0.01). In response to whether the participant felt “confident in my ability to help a person expressing agitation, stress, combativeness, hallucination, or sensory overload as a result of their progressing dementia and/or Parkinson’s disease”, there was a small change between pre- and post-intervention assessments (pre-intervention mean 7.00 (SD 3.00) vs. post-intervention 8.00 (2.50), *p* = 0.0003, d = 0.36).

### Feasibility and satisfaction

Of 52 attendees, all were able to access Zoom, navigate between the main rooms, breakout rooms, and panels, and use the chat feature to submit questions. Of all attendees, 47 were eligible for CE credit and thus pre- and post-intervention evaluations, 42 initiated the pre-intervention assessment, 39 participants completed the CE evaluation, and 35 had complete data for the pre- and post-intervention assessments and the CE evaluation.

As shown in Table 4, 100% of the 39 participants completing the CE evaluation either agreed or strongly agreed with each measure of achievement. Notably, 94.9% each strongly agreed that the teaching methods were effective for learning and that the knowledge and skills acquired in the activity were directly applicable to their professional practice.

**Table 4:** Participant evaluations of I SEE PD Home

For each statement below, please rate the level of achievement of this activity during the symposium					
***N*** ** = 39**	**Strongly** **Agree** **% (n)**	**Agree** **% (n)**	**Neither** **Agree nor** **Disagree** **% (n)**	**Disagree** **% (n)**	**Strongly** **Disagree** **% (n)**
The Facilitator(s)/Presenter(s) demonstrated content expertise	100.00 (39)	0	0	0	0
This activity met my expectations based on the stated goals and objectives	92.31 (36)	7.69 (3)	0	0	0
The teaching method(s) used were effective for learning	94.87 (37)	5.13 (2)	0	0	0
The knowledge and/or skills I have acquired from this activity are directly applicable to my professional practice	94.87 (37)	5.13 (2)	0	0	0
As a result of this activity, I am better able to collaborate with a multidisciplinary care team	89.74 (35)	10.26 (4)	0	0	0
I am better able to communicate with other members of the multidisciplinary care team as a result of what I learned in this activity	87.18 (34)	12.82 (5)	0	0	0
Because of this activity I am better able to integrate my care with other teams and team members to ensure continuous and reliable patient care	94.87 (37)	5.13 (2)	0	0	0

### Qualitative feedback

Twenty-seven of the 39 CE evaluation respondents endorsed an intent to change their practice in response to I SEE PD Home, with a sample of their verbatim, free-text responses highlighted in Table 5. Several themes emerged from these responses, including intentions to: increase interdisciplinary collaboration and disseminate new knowledge to colleagues; solicit greater involvement of and place more weight on the voice of the patient and caregiver; pay more attention to visit scheduling in relation to the patient’s function; attend to the etiology and management of sudden changes in the patient’s condition, particularly regarding urinary tract infections and orthostatic hypotension; recognize and empathize with neuropsychiatric symptoms; and apply newfound knowledge regarding pharmacologic and non-pharmacologic treatment of various symptoms. Although the chat feature was enabled throughout the entire symposium, the majority of spontaneous qualitative feedback arose during the patient and caregiver panel. Multiple participants commented that hearing directly from caregivers and patients with PD was a novel and enriching experience for them. Many of the HHA participants recognized that each PD patient responds to different stimuli or approaches and receiving validation of this from the caregivers and patients on the panel was cited as helpful by many. One participant stated “It was very educational, informative and I loved the part with real patients. Because of their feedback we can improve our services and work together as a team to provide holistic care for their loved ones.”

**Table 5:** How participants intended to change their practice after I SEE PD Home

Theme	Direct Quotations of Participants
Increased interdisciplinary collaboration	“Increasing collaboration with my team and other disciplines” “Talking with my patient’s neurologist for medication changes” “Increasing my communication with members of the patients’ care team, including the caregiver and physical therapists from outpatient clinics if patient had attended those”
Dissemination of knowledge to colleagues	“Presenting to our home health care team on what I took away from this presentation and how we can improve on our care for patients with PD”
Greater involvement of and weighting of the patient and caregiver voice	“Allowing patients and families to discuss their experiences was a helpful tool for me” “Being more aware of speaking to the patient as well as caregivers and involving the patient as much as possible” “Involving and educating family members at all times”
Attention to scheduling of visits in relation to patient function	“Taking a step back and listening to the patient and family more and being more respectful of their schedules.” “Thinking about how visits are scheduled to PD patients’ houses”
Increased awareness of etiology and management of sudden changes in patient condition	“Implementing more awareness on orthostatic hypotension with these patients as well as the importance of medication reconciliation” “Being more mindful of the incidence of orthostatic hypotension as a trigger for falls” “Practicing what I learned today, especially in recognizing an infection early on” “Focusing much more on the significance of infection, especially UTIs”
Greater recognition and empathy for neuropsychiatric symptoms	“Being more sensitive to what goes on inside the thought processes of someone experiencing Lewy body hallucinations” “Always putting myself in someone's position before making a decision and being patient because it is not who they are, but it is because of the disease they behave that way”
Pharmacologic management and medication reconciliation	“Having better knowledge of the function of levodopa… blood pressure and home care role” “Implementing more awareness on orthostatic hypotension with these patients as well as the importance of medication reconciliation”
Non-pharmacologic symptom management	“Trying tape for doorways and teaching BIG exercises” “Adding the sip-a-mug”

## Discussion

To our knowledge, this is the first medical educational intervention of its kind to specifically focus on educating home health nurses and allied health professionals about the care of people with advanced PD/PRD. Although planned prior to the SARS-CoV-2 pandemic and pivoted to a virtual format, participation in, satisfaction with, and efficacy of the virtual training were all high, in line with research on continuing education and conferences in other areas of healthcare [34, 35]. Indeed, the virtual format using Zoom® allowed for home health professionals in six different states and across different HHAs to participate and learn from each other, and the widespread availability of the platform and shared materials (in Additional file 1, 2 and 3: Appendices A-C) enhance the generalizability and ability to replicate the intervention and findings. Based on a standardized CE evaluation, all participants reported subjective improvements in knowledge and confidence regarding this patient population; objective assessments confirmed this, with a large effect on PD/PRD knowledge in particular, as demonstrated by a scale developed for this intervention. This is especially important in that it counters the likely pre-existing neurophobia in these healthcare professionals. In particular, one survey of medical, dental, OT, and SLP students found that while neuroanatomy overall was ranked the most difficult anatomic system to learn, the motor pathways and basal nuclei—the structures most involved in PD/PRD—were ranked second and fifth most difficult, respectively, out of 21 topics in the neuroanatomy curriculum [22].

While this is the first program specifically designed to educate home health professionals on PD/PRD-specific needs, similar interventions have been successful in other geriatric populations [36, 37]. Fukui and colleagues conducted a cluster randomized trial of an educational booklet and workshop on end-of-life issues for home care nurses, care managers, and care workers. Among healthcare professionals randomized to the intervention compared with control participants, active participants demonstrated improved cooperation and confidence in their ability to identify and communicate with other healthcare professionals, however there was no difference in job satisfaction between groups. Schneider and colleagues evaluated the efficacy of the Aliviado Dementia Care-Hospice Edition performance improvement program on the quality of hospice care delivered to people with dementia and their caregivers. The Aliviado program involved a two day, in-person training session for team leaders and online training for other team members. Both the team leader training and the online program demonstrated post-intervention improvements in healthcare professionals’ knowledge and confidence. However, neither of the programs above mentioned PD or PRD as part of the training despite their respective focus on end-of-life, geriatrics, and dementia, and both programs required in-person attendance. While in-person interventions are often the default, virtual models may be particularly attractive to allied health practitioners for reasons extending beyond the restrictions of the SARS-CoV-2 pandemic. Home health practitioners often reside in or near the communities they serve and may be located far from academic centers where CE and other educational programs are typically held. Access to virtual programs not only facilitates continuing education, but intra- and interdisciplinary networking opportunities [34, 35]. Particularly for home health practitioners in rural locations, they may be one of a limited number of practitioners in their area or the sole practitioner responsible for seeing a wide spectrum of patients. Providing education and resources to these more isolated providers may have disproportionate benefits for the communities they serve [38].

There were several limitations to this study. With a goal of enrolling 50 professionals in the symposium, we estimated that if each professional saw 5 PD/PRD patients per year, approximately 250 homebound PD/PRD patients could be reached with more knowledgeable, confident, and empathic providers. This was a small, uncontrolled pilot study with pre- and post-intervention data gathered within the same day, without patient-level outcome data. While longitudinal follow-up on the persistence of knowledge and attitude changes is desirable, given both the time-limited restrictions of funding and the pre-pandemic annual turnover rate of 30–65% or higher among home healthcare professionals [39, 40], we were unable to repeat participant assessments beyond the day of the symposium. This study suffers from a healthy volunteer bias, in that health professionals actively choosing to participate in I SEE PD Home may be more motivated to learn about this patient population, and both their baseline knowledge, confidence, and attitudes, along with the degree of change following the intervention, may not generalize to individuals who were offered but declined to participate in the intervention, or to the broader population of home health professionals. We anticipated that motivated participants would be more likely to participate in, gain from, and in turn serve as local champions within their respective HHAs, disseminating lessons learned to colleagues who did not choose to attend. Furthermore, to maximize recruitment within the given budgetary and time constraints, we recruited participants only from HHAs about which we had not received negative feedback from patients and families. It is plausible that home healthcare professionals in unfavorably reviewed HHAs may stand to learn and benefit the most from such an intervention, and future iterations should recruit from a broader pool of HHAs. Finally, the PD/PRD Knowledge test was created and piloted by the study team since no instrument was available to measure change in PD/PRD knowledge, with ongoing validation in a separate cohort of family caregivers.

## Conclusion

A virtually-delivered, multimodal educational intervention focused on PD- and PRD-specific issues pertinent to home healthcare professionals, incorporating general and discipline-specific content, immersive virtual reality, interactive sessions with allied health experts and with patients and caregivers, was highly successful and positively received by the participants. The virtual format allowed for simultaneous participation of home healthcare professionals across multiple states and time zones and facilitated interaction with both experts and individuals affected by PD/PRD. These features may have mitigated the neurophobia commonly contributing to poor interest in and outcomes for this population. Despite the rising global prevalence and impact of neurological conditions in general and PD/PRD specifically [2, 41, 42], and despite the frequency of referrals of individuals with advanced PD/PRD to home health practitioners [43], these critical members of the health care team have previously been overlooked in educational interventions. Future directions for I SEE PD Home include replicating and systematically testing the intervention among a broader audience of home healthcare professionals, and longitudinally assessing patient- and caregiver-level outcomes among families receiving care from providers who have completed the training versus usual care. If successful and deemed valuable by the participating home healthcare professionals, I SEE PD Home could empower participants to become knowledgeable leaders within their HHAs and communities, and agents of change for this vulnerable and expanding patient population.

## Supplementary Information

**Additional file 1. Appendix A.** Parkinson’s Disease Knowledge Survey.

**Additional file 2. Appendix B.** I SEE PD Home slides.

**Additional file 3. Appendix C.** Facilitator guides.

**Additional file 4. **I SEE PD Home Intervention Dataset combining pre- and post-intervention survey data and assessments.

## Data Availability

All data generated or analyzed during this study are included in this published article and its supplementary information files.

## Abbreviations

CBS: Corticobasal syndrome
CE: Continuing education
CORE: Community Outreach, Research, and Engagement
HHA: Home healthcare agency
HIPAA: Health Insurance Portability and Accountability Act
IRI: Interpersonal Reactivity Index
I SEE PD Home: Interactive Symposium and Education Intervention in Parkinson’s Disease Home Healthcare Professionals
LBD: Lewy Body Dementia
MSA: Multiple system atrophy
OT: Occupational therapy
PD: Parkinson’s Disease
PF: Parkinson’s Foundation
PRD: Parkinson-related disorders
PSP: Progressive supranuclear palsy
PT: Physical therapy
PTA: Physical therapy assistant
RN: Registered nurse
SD: Standard deviation
SLP: Speech-language pathology
VR: Virtual reality
