# Supplementary material for: The impact and feasibility of a brief, virtual, educational intervention for home healthcare professionals on Parkinson’s Disease and Related Disorders: pilot study of I SEE PD Home
Source: BMC Med Educ. 2022 Jun 28;22:506. doi: 10.1186/s12909-022-03430-7 (PMC9238152; doi:10.1186/s12909-022-03430-7)
Supplement: Supplementary file 1 — Additional file 1. Appendix A. Parkinson’s Disease Knowledge Survey. [file 12909_2022_3430_MOESM1_ESM.docx]

| Appendix A. Parkinson’s Disease Knowledge Survey | |
| --- | --- |
| 1. *Which of the following symptoms is*   *necessary for a diagnosis of parkinsonism?* | a. Tremor  b. Rigidity  c. **Bradykinesia**  d. Falls  e. All of the above |
| *2. Parkinson’s Disease treatments such as*  *levodopa stop working over time.* | a. True  b. **False** |
| *3. Your patient, Bob, has Parkinson’s Disease and dementia. He has been sleepier and having new falls and new hallucinations over the last 3 days. Patient and caregiver confirm that there have been no medication changes, no fever, no cough, and no urinary symptoms.*  *What is the most likely explanation?* | a. Normal course of Parkinson’s Disease;  patients can fluctuate like this.  b. **Urinary tract or other infection; urgent**  **workup with primary care doctor or**  **other healthcare provider is needed.**  c. Stroke; assess for weakness and call 911.  d. Medication non-adherence; counsel on  importance of taking medications on time. |
| *4. What are the most common causes of hospitalization in Parkinson’s Disease?* | - 1. Falls   2. Urinary tract infections   3. Hallucinations, delusions, or other neuropsychiatric symptoms   4. A & B   5. **A, B, & C** |
| *5. Your patient, Mary Ann, had Parkinson’s Disease with tremor, dyskinesias, and progressive weight loss from 120lb to 104lb. Today, her blood pressure is 170/100. She has no history of hypertension or orthostatic hypotension. Which of the following are appropriate next steps?* | **a. Repeat blood pressure measurement with a manual, pediatric cuff.**  b. Refer to primary care doctor for follow-up blood pressure check in the next week.  c. Call 911 or refer to urgent care. |
| 6. *Medication adherence is >90% in people with Parkinson’s Disease because the medications kick in/wear off more obviously and provide symptom relief (compared with baby aspirin or blood pressure medications, for example).* | 1. True 2. **False** |
| 7. *In the hospital setting, people with Parkinson’s Disease:* | 1. Are 75% more likely to die than people without Parkinson’s Disease. 2. **Have medication errors made >75% of the time.** 3. Are 75% more likely to fall in the hospital than people without Parkinson’s Disease. |
| 8. *Patients with Parkinson’s Disease are most likely to fall:* | 1. Outside of their home environment. 2. In the living room or dining room. 3. **In the bathroom.** |
| *9. You are seeing a new patient, Bill, for wound care. He sustained a scalp laceration after falling backwards on his dining room chair and hitting his head on the wall. This has been happening frequently in the last year. You notice he is very stiff and has a surprised expression on his face all the time. What is his most likely diagnosis?* | 1. Parkinson’s Disease 2. Dementia with Lewy Bodies 3. Multiple System Atrophy 4. **Progressive Supranuclear Palsy** |
| *10. Which of the following symptoms of parkinsonism are amenable to treatment?* | 1. Stiffness and slowness 2. Hallucinations and delusions 3. Drooling 4. Swallowing dysfunction 5. Sleep disturbance 6. A, C, and E 7. **All of the above** |
| Answer Key: Bolded are correct answers; Score range: 0-10, where 10 is perfect score. | |
