# Supplementary material for: The impact and feasibility of a brief, virtual, educational intervention for home healthcare professionals on Parkinson’s Disease and Related Disorders: pilot study of I SEE PD Home
Source: BMC Med Educ. 2022 Jun 28;22:506. doi: 10.1186/s12909-022-03430-7 (PMC9238152; doi:10.1186/s12909-022-03430-7)
Supplement: Supplementary file 2 — Additional file 2. Appendix B. I SEE PD Home slides. [file 12909_2022_3430_MOESM2_ESM.pdf]

RUSH

Excellence is just the beginning.

Rush University

I SEE PD Home:

Interactive Symposium and Educational Experience on Parkinson's Disease for Home Health Professionals

March 5, 2021

Jori E. Fleisher, MD, MSCE
Leslie Nan Burridge Endowed Faculty Scholar in Parkinson's Disease
Associate Professor of Neurological Sciences
Director, CurePSP Center of Care
Co-Director, Lewy Body Dementia Association Research Center of Excellence

Disclosures

Dr. Fleisher has received:

- **Research support from:**
  - NIH/NINDS
  - CurePSP
  - Feldstein Medical Foundation
  - Doris Duke Charitable Foundation Fund to Retain Clinical Scientists
  - Private philanthropic donations
- **Royalties:** UpToDate
- **Editorial Board:** AAN Brain & Life
- **Consultant:** UCB 2020-2021

All other planners, editors, faculty and reviewers of this activity have no relevant financial relationships to disclose. This presentation was created without any commercial support.

This program is supported by a Centers of Excellence CORE grant from the Parkinson's Foundation

Learning Objectives

- Discuss Parkinson's Disease and related disorders (PD/PRD), including evidence-based management to provide in-depth coverage of advanced motor and non-motor symptoms for PD/PRD.
- Recall how to recognize fall prevention and identify and perform home safety assessments to promote healthy aging-in place in PD/PRD.
- Outline the importance of medication timing and reconciliation, signs and symptoms of orthostatic hypotension and constipation, and other specific challenges for homebound PD/PRD patients.

To obtain credit you must:

- **Complete an electronic evaluation**
- **After completing the evaluation you can generate your certificate immediately.**

In support of improving patient care, Rush University Medical Center is jointly accredited by the American Nurses Credentialing Center (ANCC), the Accreditation Council for Pharmacy Education (ACPE), and the Accreditation Council for Continuing Medical Education (ACCME) to provide continuing education for the healthcare team.

ANCC Credit Designation – Nurses
The maximum number of hours awarded for this CE activity is 6.75 contact hours.

This activity is being presented without bias and without commercial support.

Rush University is an approved provider for physical therapy (216.000272), occupational therapy, respiratory therapy, social work (159.001203), nutrition, speech-audiology, and psychology by the Illinois Department of Professional Regulation.

Rush University designates this live activity for 6.75 Continuing Education credit(s).

Recording and Screens

- **We will be recording this to aid in the preparation of further symposiums**
- **We will NOT be recording during:**
  - Caregiver Panel
  - Speaker Panel
- **Please feel free to turn your screen off for duration of symposium**

Agenda

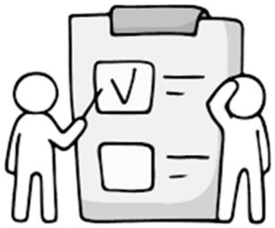

- **8:00-8:30: Welcome and surveys**
- **8:30-9:30: Pearls of Parkinson's Disease and Related Disorders: What You Need to Know**
- **9:30-10:00: Dima Lab Virtual Reality Experience – Part 1**
- ***10:00-10:15: Coffee break!***
- **10:15-11:15: Break out sessions:**
  - Nurses
  - Physical therapists, occupational therapists, speech language pathologists
- **11:15-11:45: Dima Lab Virtual Reality Experience – Part 2**
- ***11:45-12:00: Break***
- **12:00-12:15: Morning summary, review of mastery**

Agenda

- **12:00-12:15: Morning summary, review of mastery**
- **12:15-1:15: Voice of the patient & caregiver interactive panel**
- **1:15-1:30: Dima Lab Virtual Reality Experience – Part 3**
- ***1:30-1:45: Break!***
- **1:45-2:30: Detecting urgent situations in the home: what to look for**
- **2:30-3:15: Q&A panel with speakers**
- **3:15-4:00: Summary, final surveys, evaluation**

- ***It's a long day!***
- ***Eat, hydrate, stretch, check on remote learning, cuddle kids & pets as needed***

Why are we doing this?

Advanced Parkinson's Disease & Healthcare

- **Parkinson's Disease (PD) affects 1-2% of people ≥60 years old…yet…**
  - Many healthcare providers have limited training & exposure
  - Variety of motor and non-motor symptoms, variety of presentations
  - Complex medication regimens
  - Uncoordinated care & heavy reliance on family caregivers
  - High rates of emergency room visits, hospitalizations, and nursing home placement due to **falls, neuropsychiatric symptoms,** and **infections…** **many of which are preventable if detected and managed early**

Advanced Parkinson's Disease & Healthcare

MODELS OF GERIATRIC CARE, QUALITY IMPROVEMENT, AND PROGRAM DISSEMINATION

Interdisciplinary Home Visits for Individuals with Advanced Parkinson's Disease and Related Disorders

Jori Fleisher, MD, MSCE,[*†] *William Barbosa, BS,*[†] *Meghan M. Sweeney, MSW,*[‡] *Sarah E. Oyler, BSN, RN,*[§] *Amy C. Lemen, MA,*[†] *Arash Fazl, MD, PhD,*[†] *Mia K*[illegible] *Talia Meisel, BS,*[||] *Naomi Friede, BA,*[†] *Geraldine Dacpano, MPH,*[†] *Rebecca M. C*[illegible] *Alessandro Di Rocco, MD,*[†] *and Joshua Chodosh, MD, MSHS*[**††]

Original Article

Interdisciplinary palliative care for people with advanced Parkinson's disease: a view from the home

Jori E. Fleisher[1], Ellen C. Klostermann[1], Serena P. Hess[1], Jeanette Lee[1], Erica Myrick[1], Joshua Chodosh[2,3]

RESEARCH

Disease severity and quality of life in homebound people with advanced Parkinson disease

A pilot study

Jori E. Fleisher, MD, MSCE, Meghan M. Sweeney, LCSW, Sarah Oyler, RN, Talia Meisel, BS, Naomi Friede, BA, Alessandro Di Rocco, MD, and Joshua Chodosh, MD, MSHS

Correspondence
Dr. Fleisher
Jori_Fleisher@rush.edu

Neurology: Clinical Practice August 2020 vol. 10 no. 4 277-286 doi:10.1212/CPJ.0000000000000716

Abstract

Background

As Parkinson disease (PD) progresses, symptoms increase, quality of life (QoL) declines, and individuals may become homebound, often losing access to neurologic care. We aimed to determine whether facilitating expert *in-home* care could improve our understanding of disease progression, treatment options, and unmet needs in this vulnerable population, and whether such a model could mitigate decline in QoL.

Methods

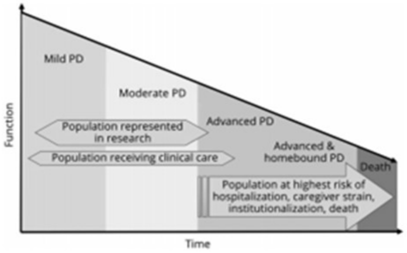

Fleisher J, et al. J Am Geriatr Soc. 2018 Jul;66(6):1226-1232.
Fleisher JE, et al. Neurol Clin Pract. 2020 Aug;10(4):277-286.
Fleisher JE et al. Ann Palliat Med. 2020 Feb;9(Suppl 1):S80-S89.

Sustainability…

Specialized outpatient team

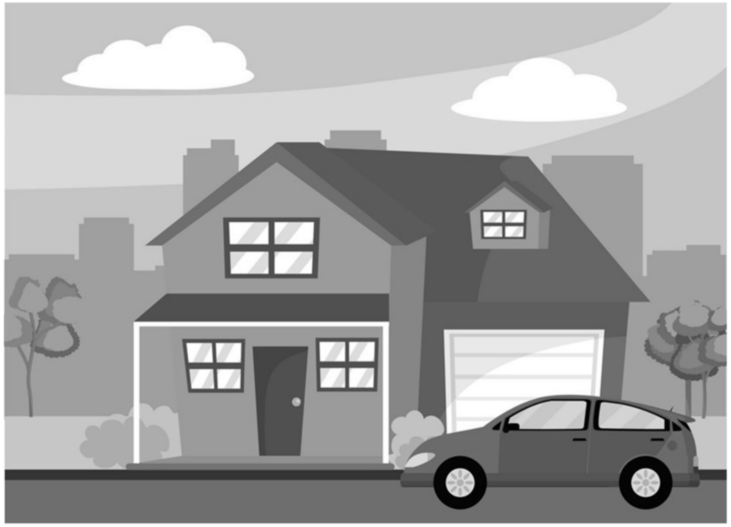

Sustainability…

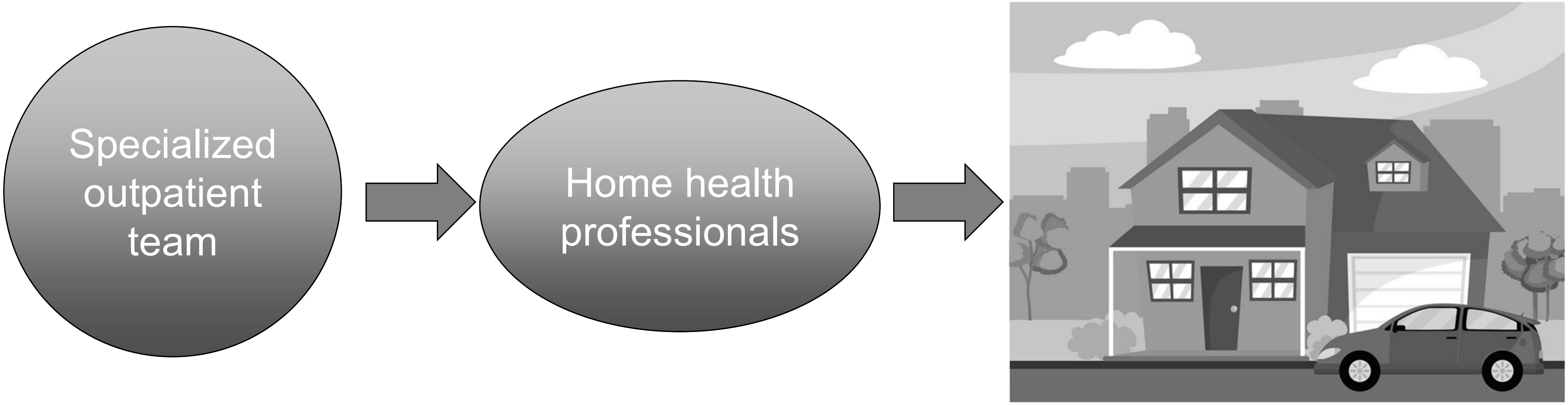

First, quick surveys

- **Navigate to survey link**

1. **Answer brief anonymous questions that will help us connect pre surveys with post surveys: First initial of first name, color of first car, childhood home street name**
   - For example, *J Red Sesame*
2. **Demographic questions (age, gender identity, race, ethnicity, discipline, years of experience, experience with PD)**
3. **Interpersonal reactivity index**
4. **Two brief PD knowledge tests**

- **Once you're done, please send a chat message to Serena Hess: First name, last initial, DONE**

Survey link

Provided during presentation

Questions? Onto Next Presentation!

RUSH

Excellence is just the beginning.

Rush University

Pearls of Parkinson's Disease and Related Disorders: What You Need to Know

March 5, 2021

Jori E. Fleisher, MD, MSCE
Leslie Nan Burridge Endowed Faculty Scholar in Parkinson's Disease
Associate Professor of Neurological Sciences
Director, CurePSP Center of Care
Co-Director, Lewy Body Dementia Association Research Center of Excellence

Overview

- Parkinson's? Parkinsonism?
  - Typical vs. atypical parkinsonism
- Motor symptoms & approaches
- Non-motor symptoms & approaches
- Complications of advanced PD
- Atypical parkinsonism

Typical vs. Atypical

Epidemiology of PD

- **First described by James Parkinson in 1817!**
  - **"An Essay on the Shaking Palsy"**
- 2nd most common neurodegenerative disease
- Prevalence increases with age:
  - 1-2% $\geq$ 60 yrs
  - 3-5% $\geq$ 80 yrs
- Affects > 5 million worldwide
- Male to female ratio: 1.46:1
- Under-diagnosed in minority populations
- Unknown environmental/lifestyle risk factors
  - Pesticide exposure increases risk
  - Caffeine, cigarette smoking, NSAID use, gout *might* decrease risk

Case 1

- 55 year-old right-handed male presents with 1 year of R shoulder pain and R arm stiffness
- Wife says he doesn’t swing his arm when they take walks, and R hand shakes while walking.
- Exam: Decreased facial expression with few blinks. Slow finger taps and rapid alternating movements bilaterally, R>L, that decrease in amplitude. Stiff right arm when walking with low-frequency pronation-supination tremor of R hand

CASE 1 VIDEO

Parkinsonism vs. Parkinson's Disease

<u>Parkinsonism?</u>

- Bradykinesia
  (slow, small movements)

\+ $\geq$1 of:

1. Rigidity
   (muscle stiffness)
2. Tremor
   (usually at rest and starting on one side)
3. Imbalance/posture changes

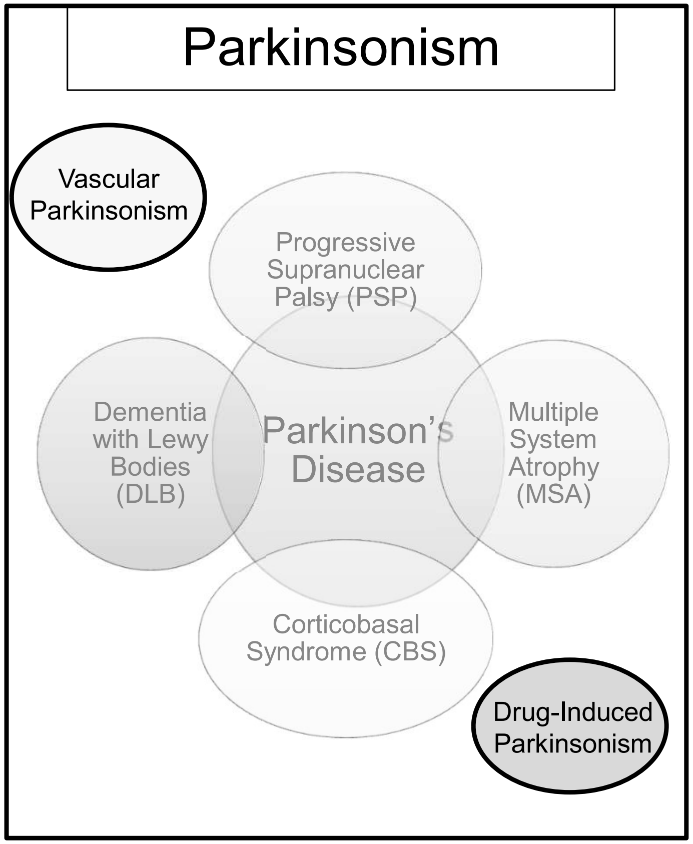

What's Typical for Idiopathic PD?

CARDINAL FEATURES:
- Bradykinesia + $\geq$1 of the following:
  - Rigidity, tremor (resting), postural instability

SUPPORTIVE FEATURES:
- Unilateral onset, asymmetric disease
- Levodopa responsiveness, lasts $\geq$5 years
- Levodopa-induced dyskinesia
- Clinical course $\geq$10 years

PRE-MOTOR FEATURES:
- Olfactory loss
- REM Sleep Behavior
- Constipation

➢ ***30% of PD patients NEVER have a resting tremor***

➢ ***Tremor often does NOT respond to levodopa!***

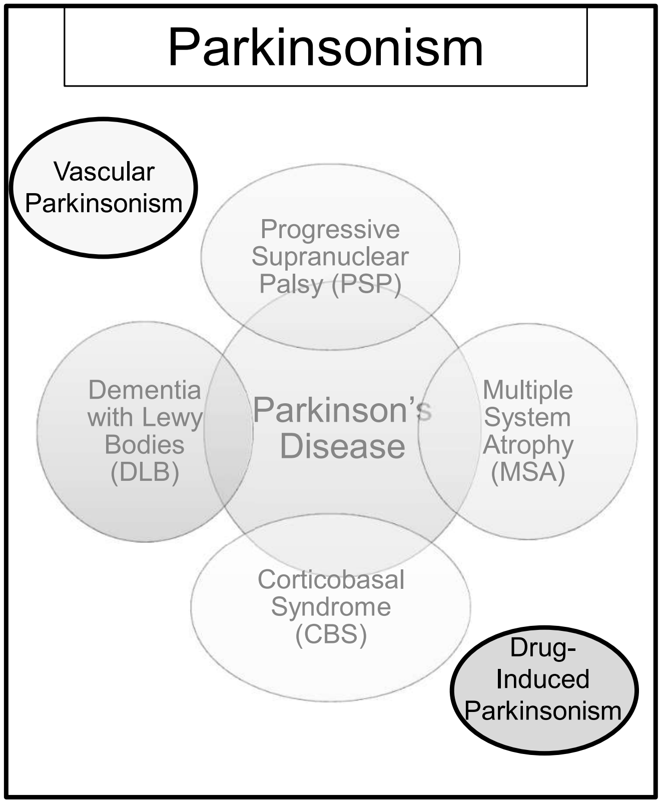

What’s *Atypical* for Idiopathic PD?

- Early decline in cognition and fluctuating cognitive performance
- Early recurrent, complex visual hallucinations
- Early and prominent autonomic dysfunction: urinary incontinence, erectile dysfunction, constipation, orthostatic hypotension
- Gait and/or limb ataxia and dysarthria
- Levodopa-resistant akinetic-rigid syndrome
- Vertical supranuclear gaze palsy

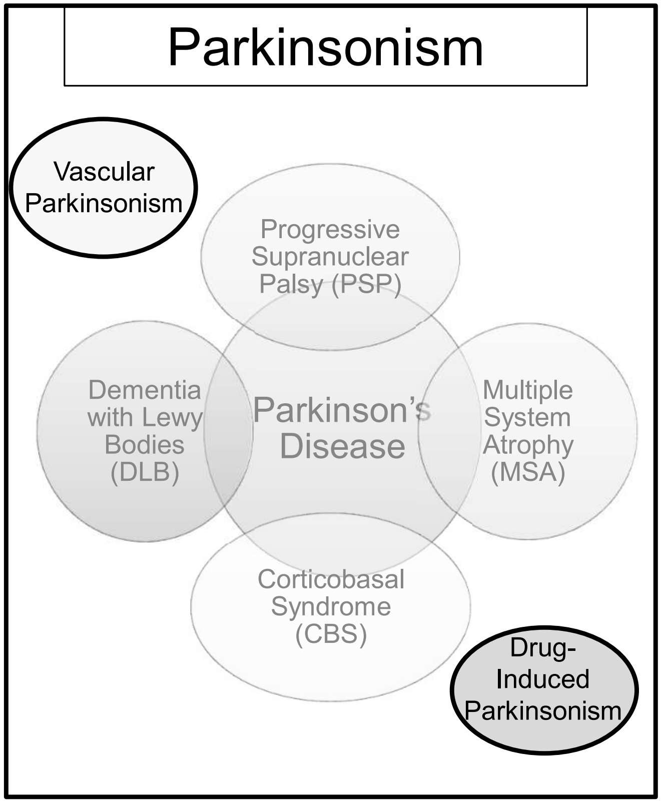

What's *Atypical* for Idiopathic PD?

- Falls within the first 3 years of symptoms
- Pseudobulbar affect
- Apraxia, alien limb phenomenon, cortical sensory changes
- Prolonged or contemporaneous exposure to dopamine-receptor blocking agents
- Vascular risk factors and sudden stepwise declines in motor function and/or cognition

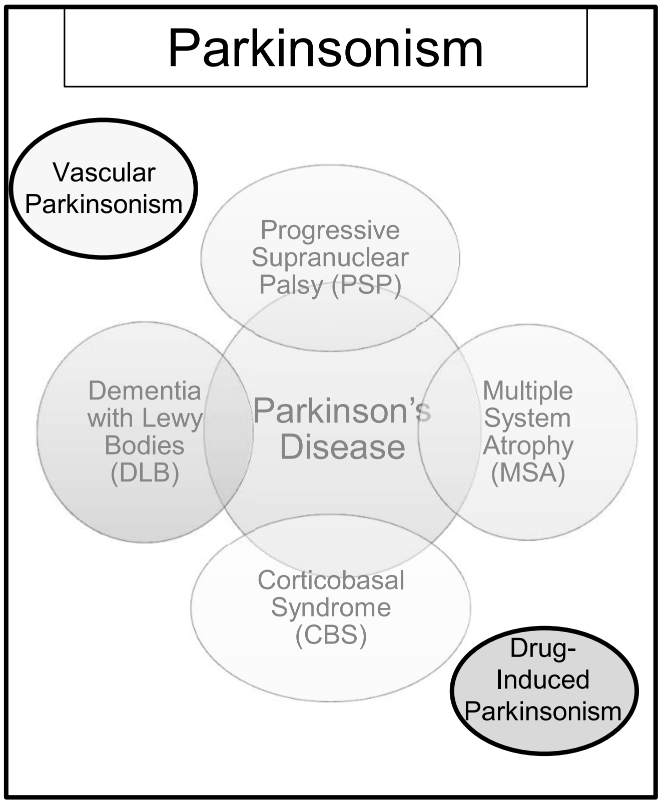

Stages of Parkinson's Disease

- "Advanced PD":
  - Hoehn & Yahr 4-5, sometimes 3
  - At least 1 of the following:
    1. Disability limiting independence
    2. Loss of postural reflexes or severe postural deformity
    3. Freezing phenomenon
    4. Levodopa complications that become the focus of treatment

Hoehn and Yahr Scale
1.0: unilateral symptoms
2.0 Mild, bilateral involvement, balance is normal
3.0: Mild-moderate bilateral involvement, balance is impaired
4.0: Severe, can ambulate unassisted
5.0: Wheelchair- or bedbound unless aided

Savica R, et al. *Handbook of clinical neurology.* 2016;138:153-158.
Goetz CG, et al. *Movement disorders : official journal of the Movement Disorder Society.* Sep 2004;19(9):1020-1028.
Fahn S, et al. *Principles and practice of movement disorders.* 2nd ed. Edinburgh ; New York: Elsevier/Saunders; 2011

Stages of Parkinson's Disease

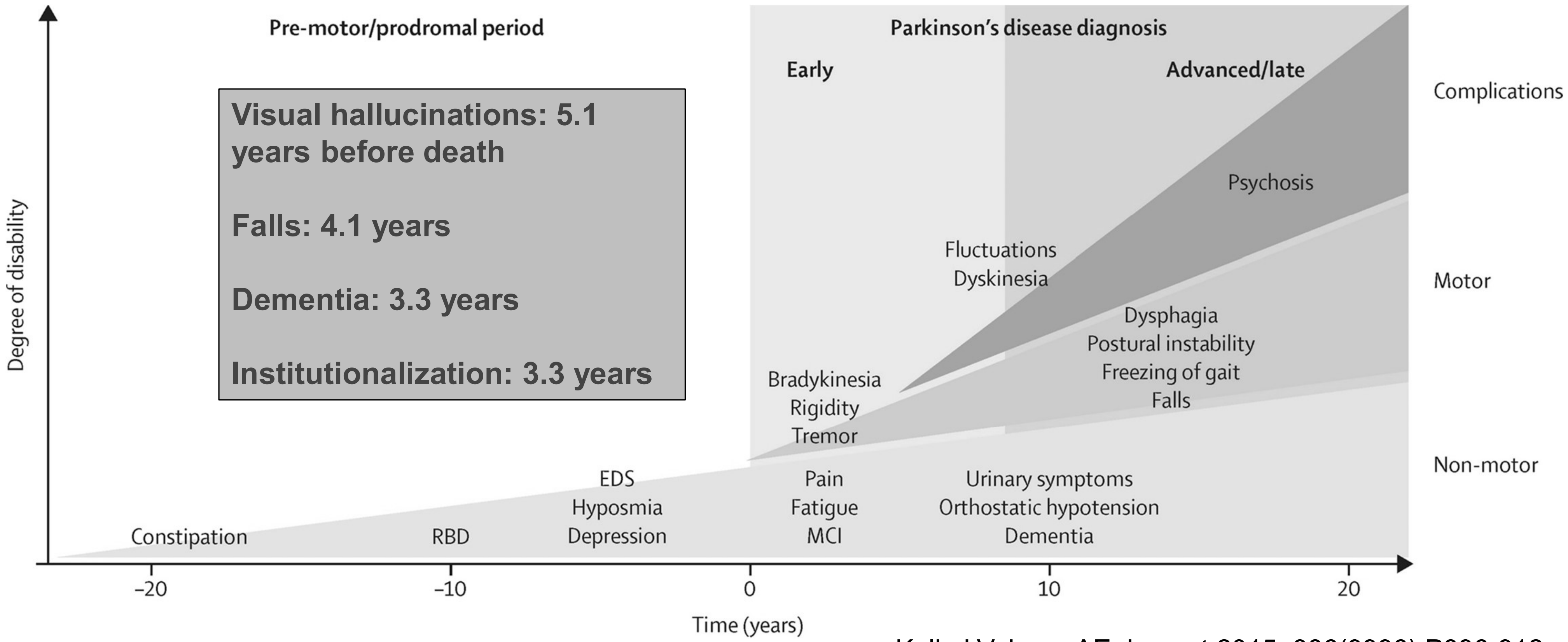

Visual hallucinations: 5.1 years before death

Falls: 4.1 years

Dementia: 3.3 years

Institutionalization: 3.3 years

Kalia LV, Lang AE. Lancet 2015: 386(9996):P896-912.
Fabbri M, et al. J Parkinsons Dis. 2020;10(s1):S75-S83.

Motor Symptoms

Early PD

- Cardinal motor features predominate
- Dopaminergic medications provide excellent relief of motor symptoms
  - Tremor may be exception
- Function independently in their occupation and social activities
- Medication effects last until next dose
- Relatively consistent effect from medications.

Moderate PD

- Motor fluctuations
- Non-motor symptoms more prominent
  - Depression, anxiety
  - Constipation
  - Orthostatic hypotension
  - Urinary urgency, frequency, nocturia, incontinence
  - Erectile dysfunction

http://www.medscape.org/viewarticle/701955

Case 2

Case 2 VIDEO

Evolution of Fluctuations

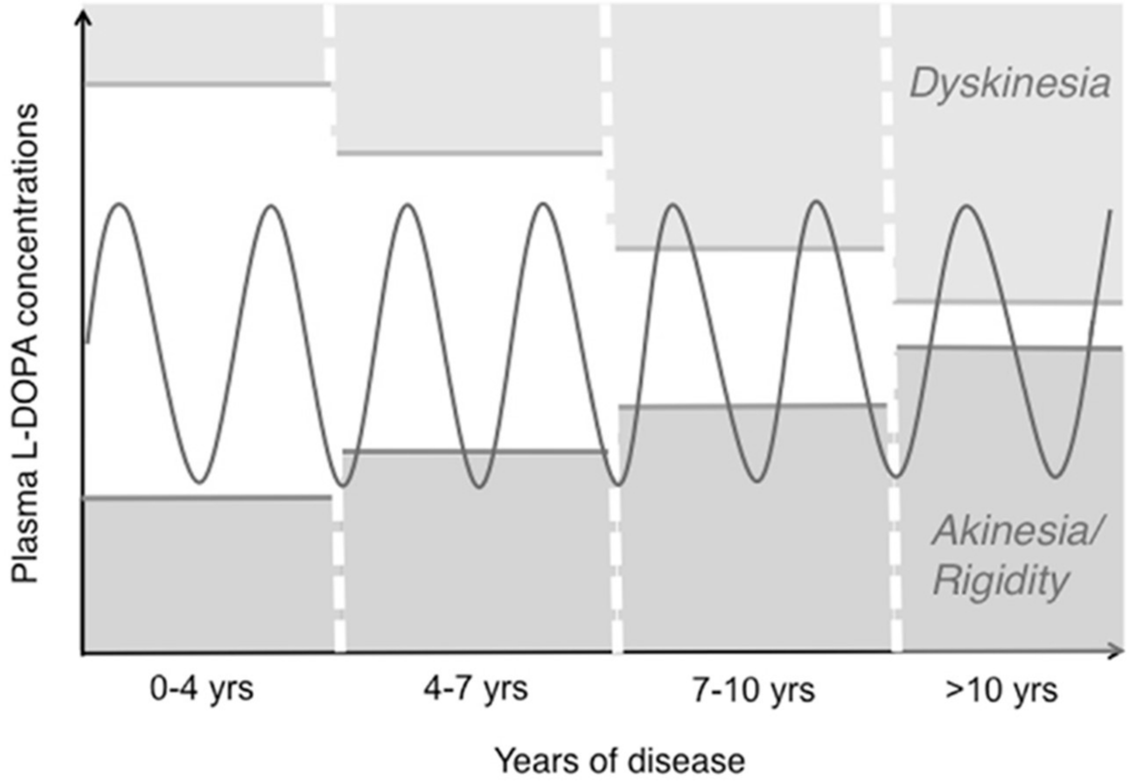

Cenci MA. Presynaptic Mechanisms of l-DOPA-Induced Dyskinesia: The Findings, the Debate, and the Therapeutic Implications. Front Neurol. 2014 Dec 15;5:242.

Managing Fluctuations

- Increase frequency of levodopa doses / decrease time between doses
- Add longer-acting dopaminergic stimulation
- Enhance duration of each levodopa dose
  - COMT inhibitor (entacapone, or switch to carbidopa-levodopa-entacapone)
- Consider continuous dopaminergic stimulation with levodopa intestinal gel
- ***Any additional dopaminergic stimulation is going to* worsen *hallucinations, confusion, orthostasis, dyskinesias***

Freezing of Gait

- Sudden, episodic gait dysfunction (OFF)
  - Extremely short steps or complete inability to move in any direction
  - Start hesitation → transitions & turns→ generalized
  - Associated with falls, poorer prognosis, decreased survival
- Treatment*
  - Adjust levodopa
  - Consider MAO-B or AChE inhibitor
  - *Physical therapy*
  - *Treat anxiety*
  - *Consider subclinical orthostatic hypotension*

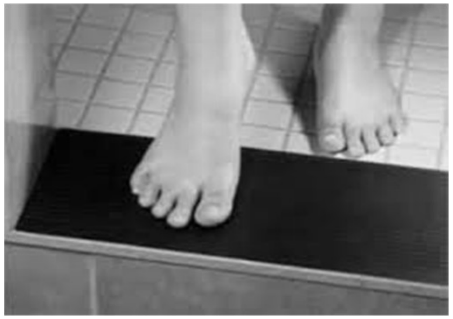

Cucca A, et al. Neurodegener Dis Manag. 2016 Oct;6(5):431-46.
Jankovic J. Gait disorders. Neurologic clinics 33(1), 249-268 (2015)
Smulders, Katrijn et al. Parkinsonism & related disorders vol. 31 (2016): 3-13.

Falls

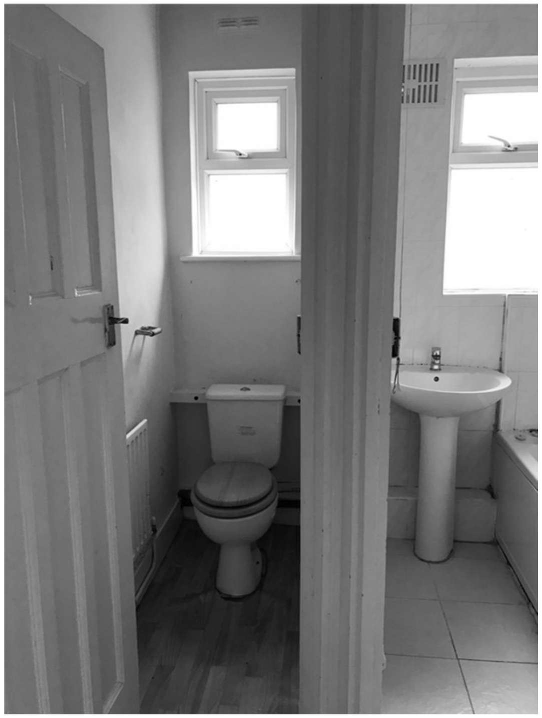

- Identify contributing factors
  - Wearing-off
  - Freezing of gait
  - Imbalance
  - Impulsivity
  - Orthostatic hypotension
- Identify setting
  - Absence of or inappropriate assistive device
  - Tight spaces—bathrooms, kitchen
  - Multitasking, particularly on stairs
  - Poor home safety
- Plan: Physical therapy & home safety evaluation

Approach to treatment…

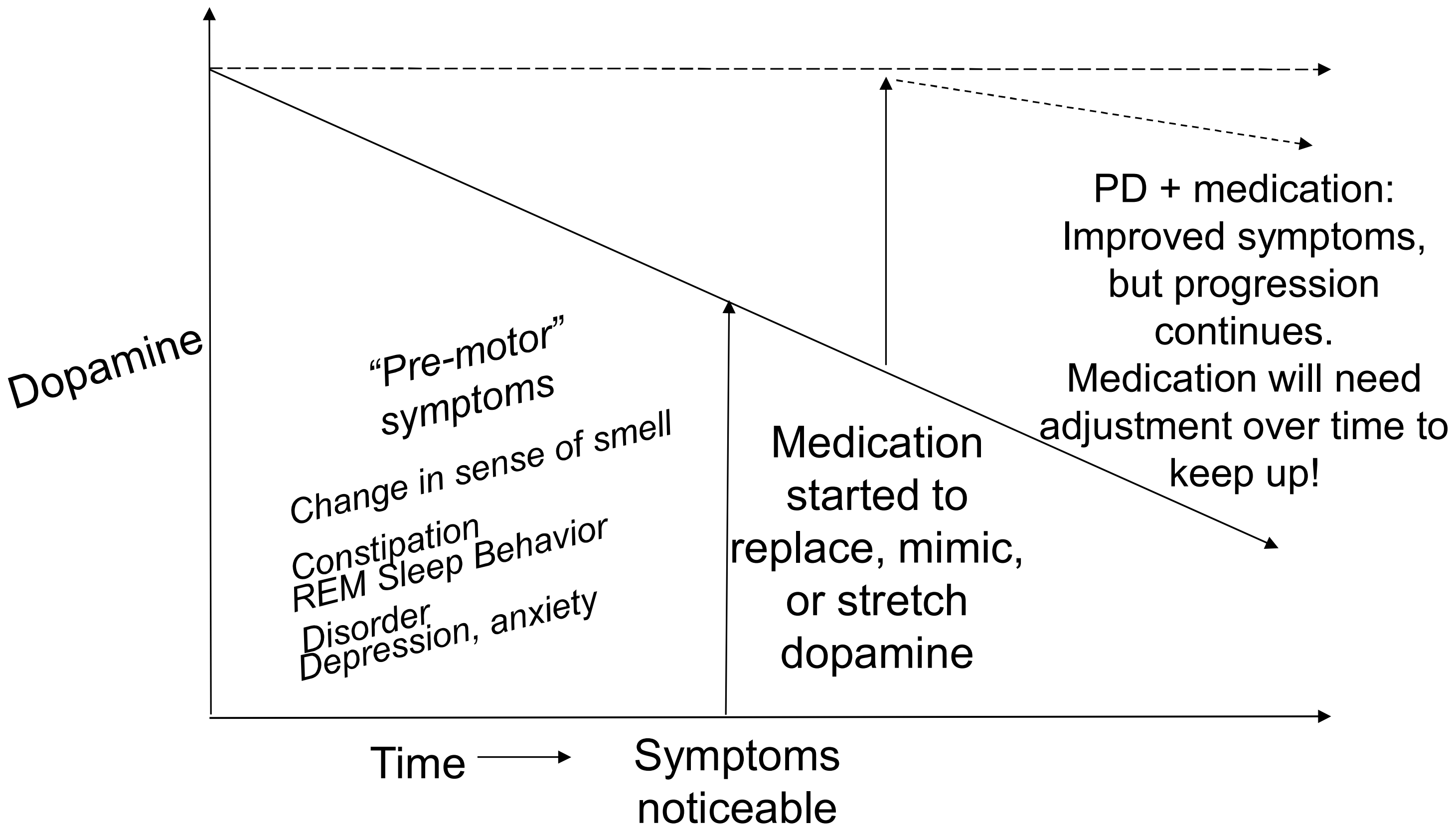

Or… the bandage combo pack analogy…

Parkinson's Disease: Treatment

- Tenets of therapy
  - All treatments are symptomatic
    - no FDA/AAN-approved neuroprotective agents…
  - Start tx when symptoms interfere with ADLs, job performance
  - Encourage patients to remain active & mobile
  - Choice depends on predominant sx, age, severity, comorbidities, side effects…so *individualize therapy!*
- Menu Options
  - Levodopa/carbidopa
  - Dopamine agonists
  - MAO-B inhibitors
  - COMT inhibitors
  - *Anticholinergics*
  - *Amantadine*
  - Deep brain stimulation

Figure. Theoretical Models of Levodopa Use and Associated Disability

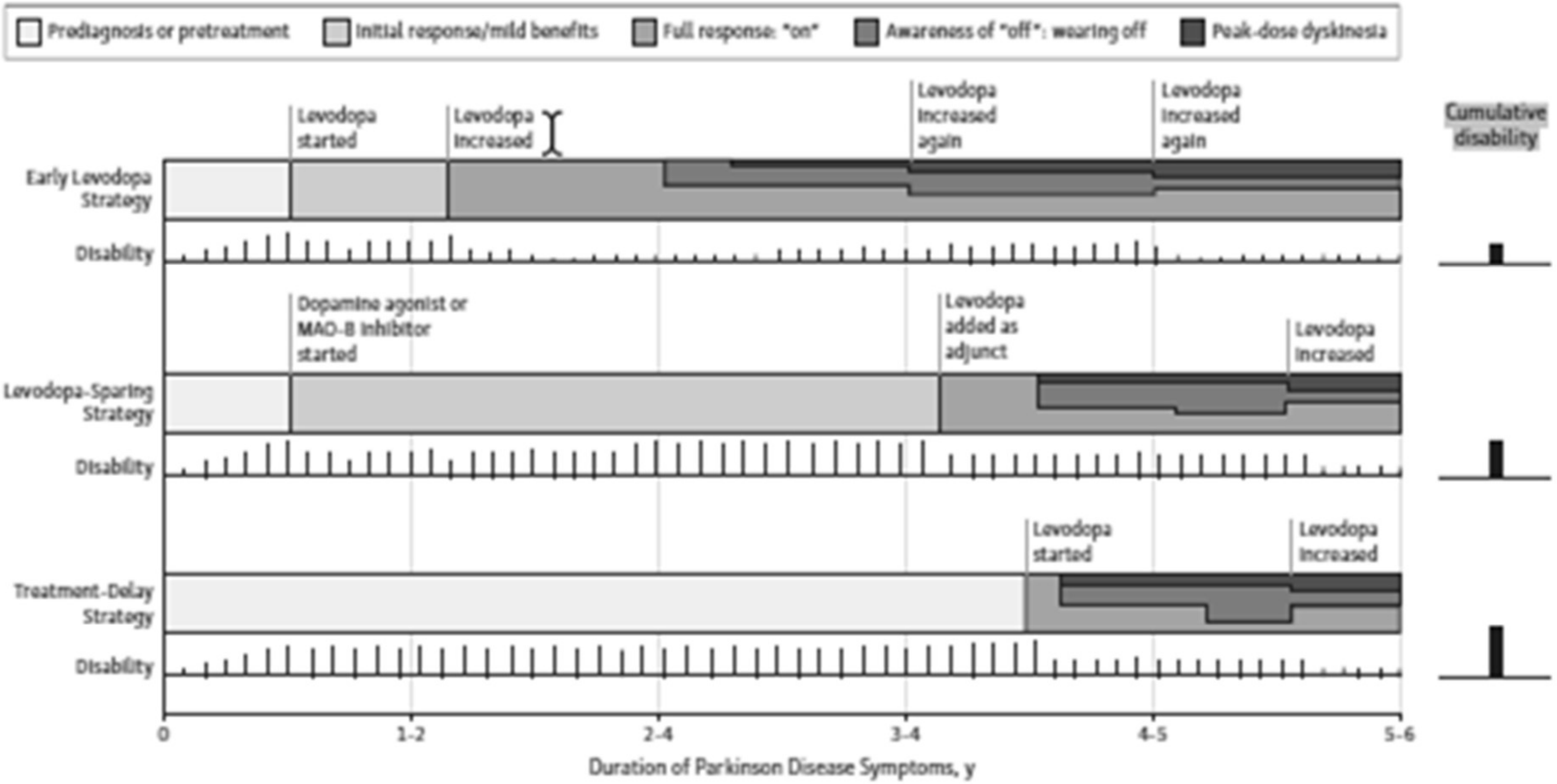

Early levodopa initiation induces a larger magnitude of response and overall lower cumulative disability compared with levodopa-sparing strategies (dopamine agonists pramipexole, ropinirole, and rotigotine; monoamine oxidase B [MAO-B] inhibitors selegiline and rasagiline), despite earlier appearance of motor fluctuations. While increased levodopa doses to optimize motor function worsen peak-dose dyskinesia, overall disability remains more closely related to "off" periods. Note that the awareness of "off" (wearing off) is always preceded by an appreciation of full response or "on" state.

Espay AJ and Lang AE. JAMA Neurol, June 2017, 74(6).

Non-Motor Symptoms

Sialorrhea and rhinorrhea

- First-line: sialogogues
  - Consider glycopyrrolate
- Botulinum toxin injections to parotid & submandibular glands

Comparing the four major botulinum toxins.

Toxins	Brand Name	Indication	Company	Clinical Trial	Dosage	Side-Effect/Cons
**Botulinum toxin A**						
IncobotulinumtoxinA	Xeomin	FDA-approved for chronic Sialorrhea	Merz Pharmaceuticals (Germany)	SIAXI	100 U	Dry mouth, dysphagia [28]
OnabotulinumtoxinA	Botox	No FDA approval for Sialorrhea	Allergan US		-	Unknown
AbobotulinumtoxinA	Dysport	No FDA approval for Sialorrhea	Ipsen (France)		-	Unknown
**Botulinum toxin B**						
RimabotulinumtoxinB	Myobloc	FDA-approved for chronic Sialorrhea	Myobloc (USA)	Isaacson et al.	2500 U and 3500 U	Dry mouth, dysphagia, and dental caries [29]

Seppi K, et al. Mov Disord. 2011;26(0_3):S42-S80.
Isaacson J, et al. Toxins vol. 12,11 691. 31 Oct. 2020.

Dysphagia

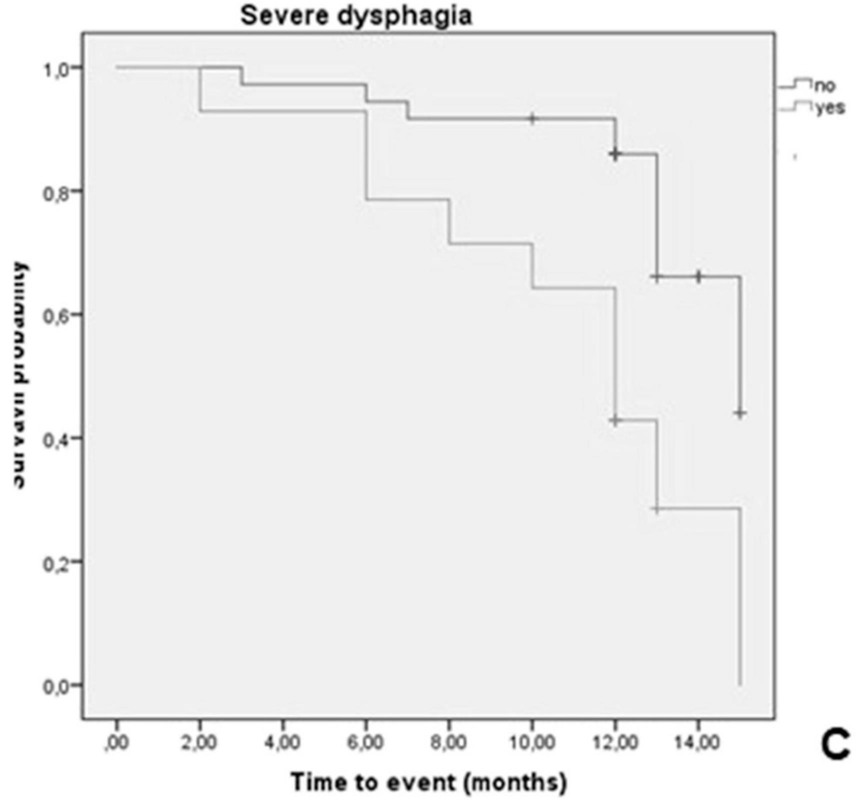

Severe dysphagia at baseline predicts death, HY 5, or institutionalization at 12 months (HR 2.3, 95% CI 1.12-4.4, p = 0.01)

Fabbri M, et al. Parkinsonism Relat Disord. 2019 Jul;64:73-81.

Dysphagia

- SLP, swallow evaluation
  - Mobile swallow units and evaluations are possible
  - Virtual speech and swallow therapy
- Altered diets
  - Thickening liquids can help
  - Cut food into small, manageable pieces
  - SMALL sips, not big gulps
  - SMALL bites, not big mouthfuls
- Maintaining oral hygiene

Table 3 Characteristics of oropharyngeal dysphagia in Parkinson's disease and Parkinsonian syndromes.

Disorder	Characteristics
Parkinson's disease	Difficulty with bolus manipulation and control[27,87,88]
	Xerostomia[27]
	Delay in initiation of pharyngeal swallow[27,87]
	Residue in pharynx[27,87]
	Abnormal airway somatosensory function[27]
	Decreased upper esophageal opening and relaxation [88]
	Silent aspiration[87]
PSP	Difficulty with bolus manipulation and control[89]
	Premature loss of bolus into pharynx[89]
	Residue in valleculae and pharynx[89]
MSA	Slow bolus transfer from oral to pharyngeal cavity[90]
	Prolonged hold of bolus in oral cavity[90]
	Inccordination of tongue[90]
	Aspiration[90]

Walshe M. Journal of Gastroenterology and Hepatology Research 2014;3(10)

Unintentional Weight Loss

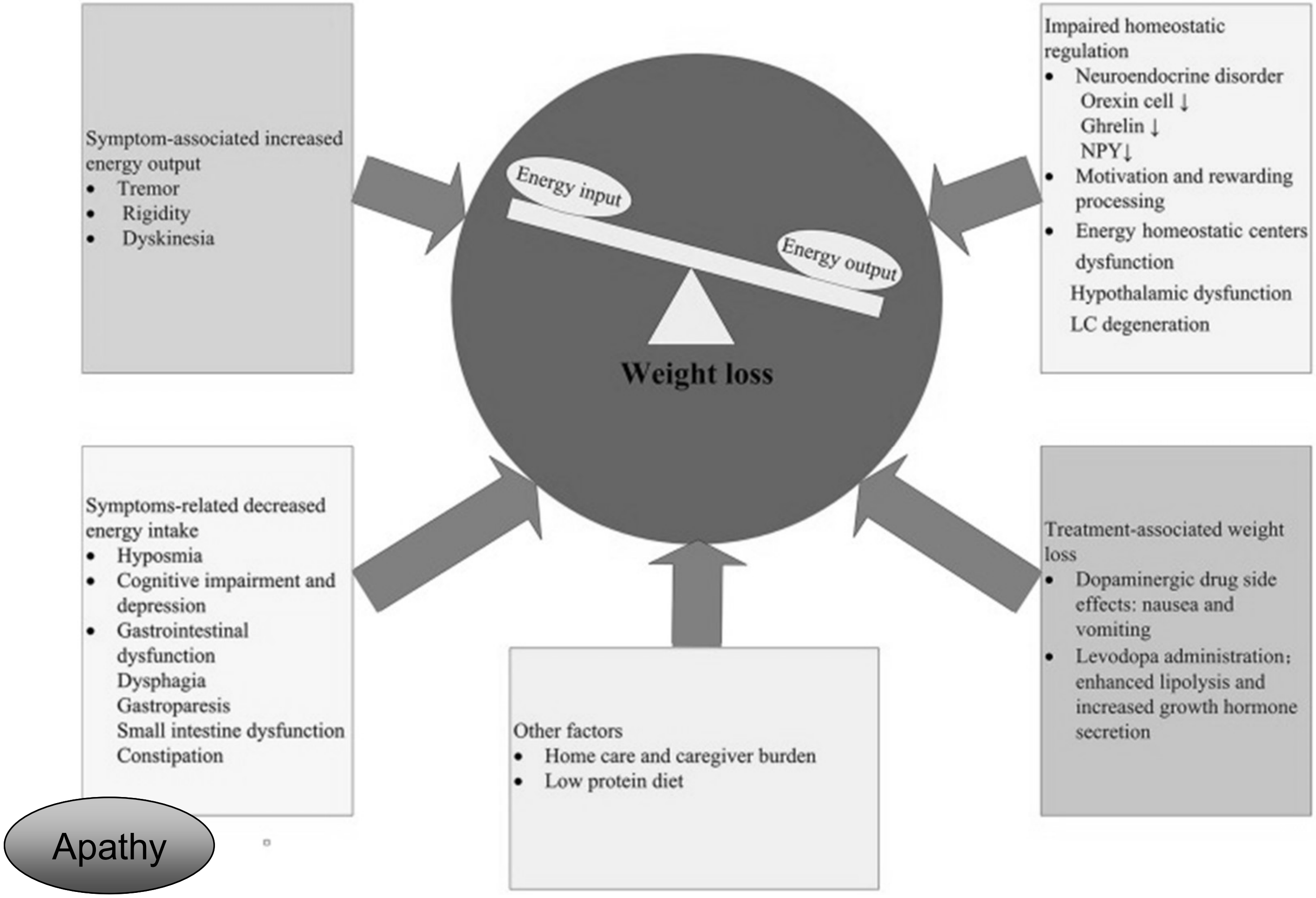

Ma, Kai et al. Frontiers in aging neuroscience vol. 10 1. 19 Jan. 2018.

Unintentional Weight Loss

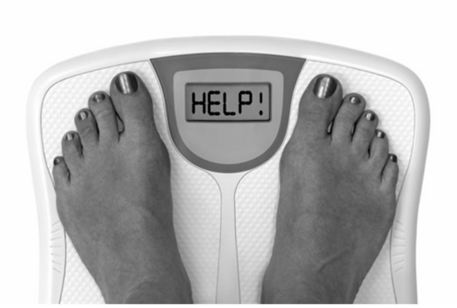

- Middle or early advanced stages:
  - – Age-appropriate screenings
  - – Exclude other treatable causes
- Dental evaluation, swallow evaluation
- Review medications
- Nutritionist consultation
  - – Small, frequent meals with added seasoning
  - – High calorie-density foods
    - Nutritional shake + peanut butter + ice cream
- Counsel family regarding anosmia, anorexia

Ma, Kai et al. Frontiers in aging neuroscience vol. 10 1. 19 Jan. 2018.
Hopkinson JB. Eur J Oncol Nurs. 2016 Feb;20:87-96.

Constipation

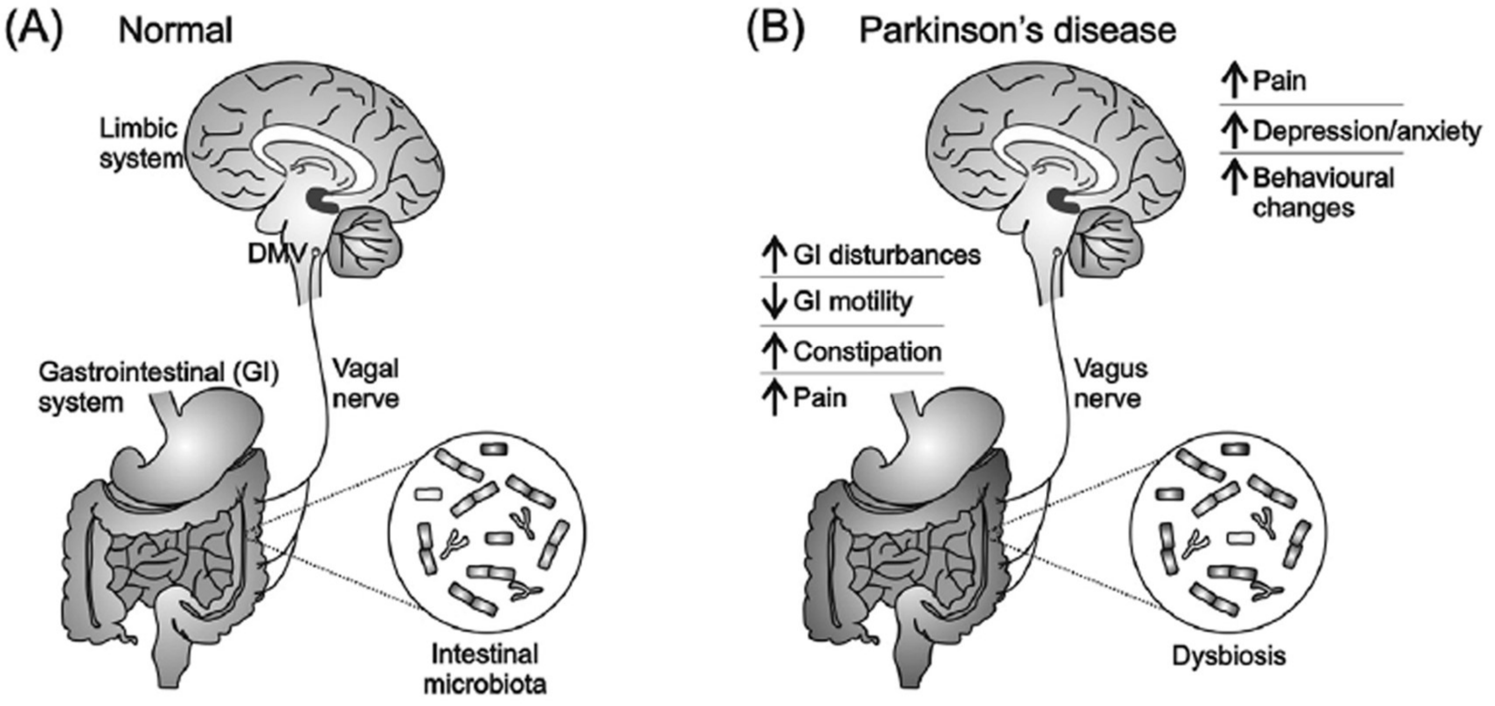

Credit: Felice VD et al./Parkinsonism Relat Disord 2016

Armstrong MJ, Okun MS. Diagnosis and Treatment of Parkinson Disease: A Review. JAMA. 2020 Feb 11;323(6):548-560.

Urinary Dysfunction

- Nocturia
  - Waking up *to* urinate vs. waking up…and urinating?
- Urinary urgency, frequency, incontinence
  - 1st step: Urinary issue vs. motor issue?
  - Urinary: Bladder training; Solifenacin, darifenacin, or mirabegron; consult urology

Palma JA, et al. Mov Disorders 2018:33(3):372-390.

Orthostatic Hypotension

- Review meds
  - *Still* hypertensive?
  - Long-term risks and benefits of anti-HTN vs. immediate risks of hypotension
  - *Check BP with an appropriate cuff!*
- Common, underreported cause of falls, fatigue, immobility

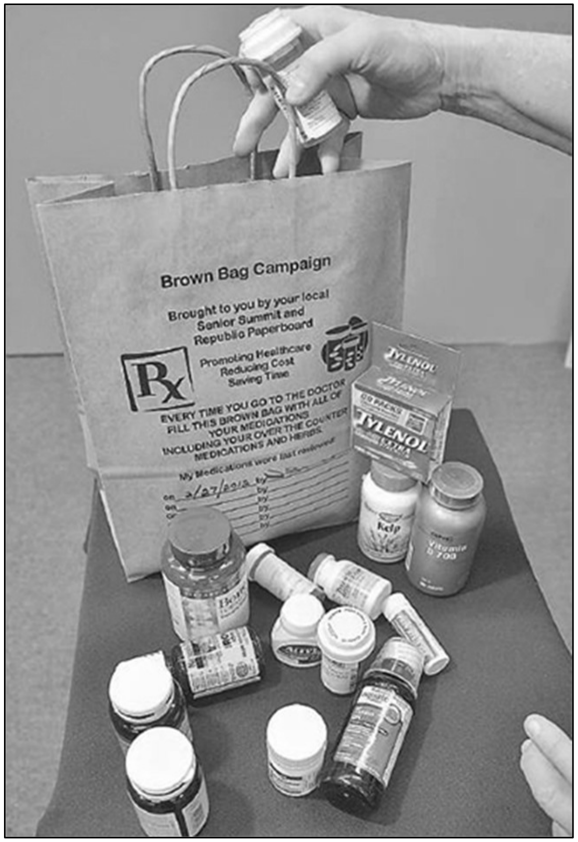

Palma JA, et al. Mov Disorders 2018:33(3):372-390.
Biaggioni I. Am J Hypertens. 2018 Nov 13;31(12):1255-1259.

Sleep Disorders

- Insomnia:
  - Depression, anxiety, poor sleep hygiene, restless legs syndrome, dystonia, pain, nocturia, nightmares, sleep apnea
  - Treatments: melatonin, mirtazapine, trazodone
  - *Sleep hygiene education for patient & caregive[illegible]*
- REM Behavior Disorder:
  - Melatonin (3-12mg)
  - Low-dose clonazepam (0.5-2mg)
- Home safety:
  - Yoga/gym mats around bed
  - Bed rails, padding, bed alarms
  - Move nightstands away from bed or cushion corners

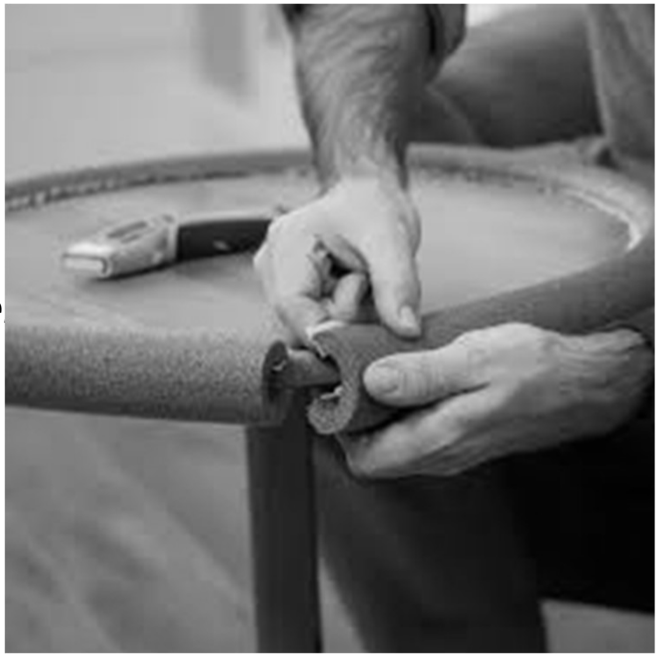

Depression

- **#1 predictor of quality of life in PD**
- 30-40% cumulative prevalence
  - *NOT* a reaction to the diagnosis
  - Related to serotonergic & noradrenergic changes
- Non-medication treatments:
  - Social involvement, support groups
  - Exercise
  - Cognitive-behavioral therapy
- Medication
  - SSRIs, SNRIs > TCAs

Barone P, et al. *Mov Disord*. 2009;24(11):1641-1649.

Anxiety

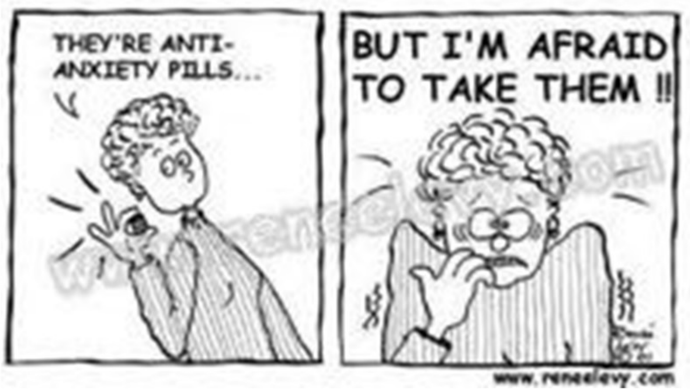

- Up to 40% cumulative prevalence
- Symptoms
  - Loss of confidence, fear of social occasions & public speaking
  - Generalized anxiety state
  - On/off anxiety states
- Treatment options
  - Medication-related on & off anxiety: PD medication adjustments
  - Psychotherapy
  - Generalized anxiety: Anti-depressants
  - Anxiolytics: clonazepam, lorazepam, buspirone

Dementia

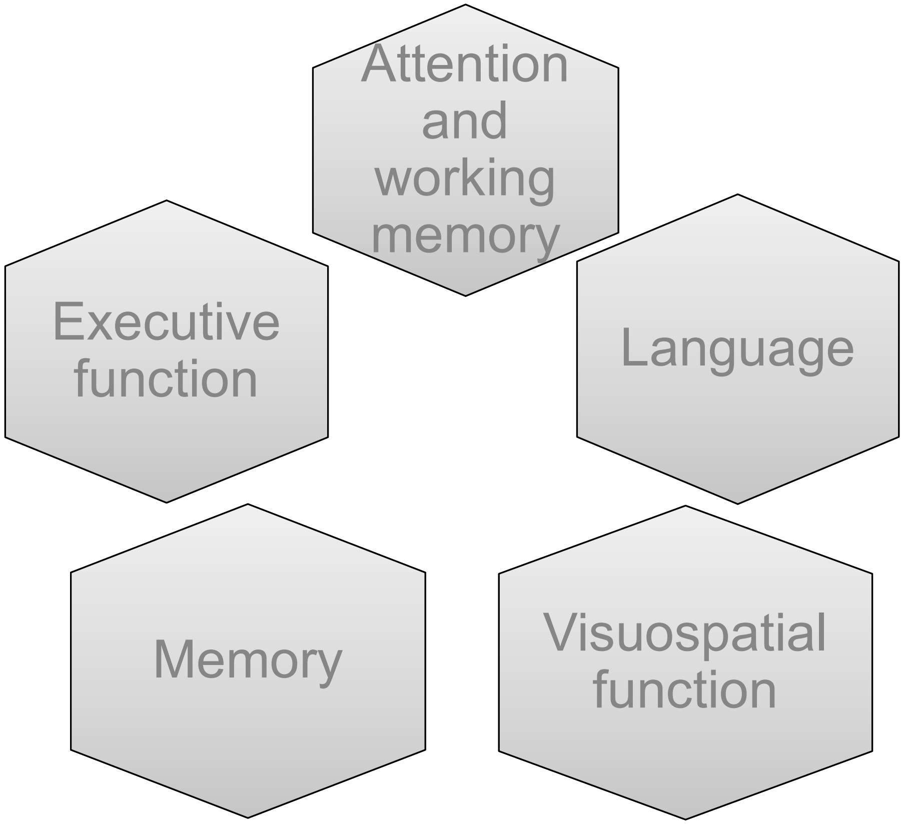

- 30% point & 70-80% cumulative prevalence
  - Comorbid AD in 30%
- Cognitive fluctuations, visual >> auditory hallucinations, depression, sleep disturbance distinguish this from AD
- Dementia does *not* mean the person is immune to depression & anxiety

Psychosis and Hallucinations

- Up to 60% prevalence
- Hallucinations:
  - **Illusion**: mistaking real object for something else
  - **Sensory illusions**: 'passage' hallucination, sensing a presence
  - **Visual hallucinations**: evolve from simple, non-threatening, preserved insight to complex, frightening, constant, limited insight
- Delusions:
  - **Paranoia**: generalized or specific; delusional jealousy
  - **Reduplicative paramnesias**: Capgras syndrome and others
    - ***Ask and you shall receive…***

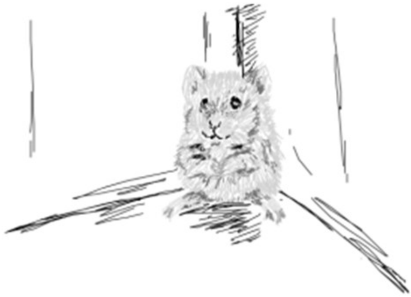

https://doodleordie.s3.amazonaws.com/d/aHHRlg55/eHkUCyd6M.png

Cannas A, et al. Neurol Sci. 2017;38(2):225-231.

Treatment Approach

- Simplify medication regimen to balance motor and cognitive difficulties
- Maintain a structured, familiar environment
- Maintain a regular sleep-wake cycle & circadian rhythm
- Individualized daily schedule
- Caregiver and family education & support

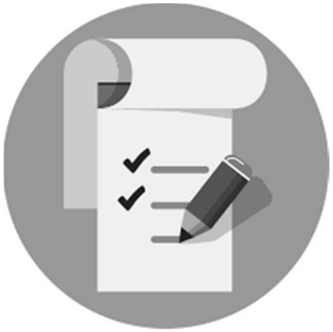

Treatment Approach

- To treat cognition:
  - Acetylcholinesterase inhibitors: Rivastigmine, donepezil
  - NMDA antagonist: Memantine
- To treat psychosis:
  - *Reduce unnecessary medications*
  - Antipsychotics:
    - Quetiapine—Low doses to avoid worsening parkinsonism
    - Clozapine—Requires weekly blood monitoring for agranulocytosis
    - Pimavanserin—No long-term data yet; less sedating, may transiently worsen symptoms before improvements appreciated

Seppi K, et al. *Mov Disord.* 2011;26(0_3):S42-S80.

Putting It All Together

- Cross-sectional study of 82 home-dwelling patients with PD or related disorders, HY 3-5
  - >80%: problems using legs/walking; fatigue; pain; daytime sleepiness
  - >70%: sialorrhea; trouble using arms and hands
  - >60%: difficulty communicating; spasms, cramps, or stiffness; constipation
  - >50%: urinary dysfunction; dysphagia, dyspnea
  - 42%: fall in the past 2 weeks
  - 31%: bowel incontinence
  - 26%: poor appetite
  - 26%: hallucinations

Saleem TZ, et al. Palliative Medicine 2012; 27(8):722-731.

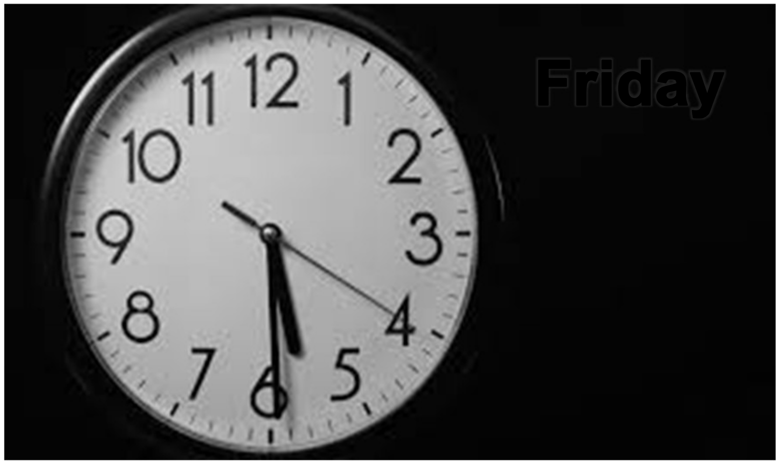

Complications and Sudden Changes in Advanced PD

Sudden Changes in Advanced PD

- Abrupt changes in mobility – diminished efficacy of medications, increased OFF time, increased freezing of gait
- Abrupt changes in cognitive status
- New or significantly worsened hallucinations or delusions

Sudden Changes & Culprits

- Abrupt changes in mobility – diminished efficacy of medications, increased OFF time, increased freezing of gait
- Abrupt changes in cognitive status
- New or significantly worsened hallucinations or delusions

- Exclude reversible causes
  - **UTI, UTI, UTI**
  - **COVID,** URI, (aspiration) pneumonia
  - Other infectious causes (teeth, seat, feet)
  - Impaction or bowel obstruction
  - Metabolic derangements, dehydration
  - Medication errors or new medications

Complications of Advanced PD

- Subclinical infections
  - Urinary tract infections account for 9% of PD hospital admissions in one study
- Aspiration pneumonia
  - PNA accounts for 11% of PD hospital admissions
  - Advanced PD associated with 2.5-fold increased risk of hospitalization for influenza or PNA
- Falls, fractures
  - 14% of PD hospital admissions
- Impaction, bowel obstruction

Hassan A, et al. 2013. *Parkinsonism Relat Disord* 19, 949-954.
Gerlach, OH et al. 2011. *Mov Disord*, 26, pp. 197-208.

Don’t wait!

- ***If you were suddenly seeing new visual hallucinations of intruders in your home… would YOU notice the color of your urine?***

- **By the time patients develop “typical” signs and symptoms, it may be too late**

Should these patients get the COVID vaccine?

YES

Atypical parkinsonism

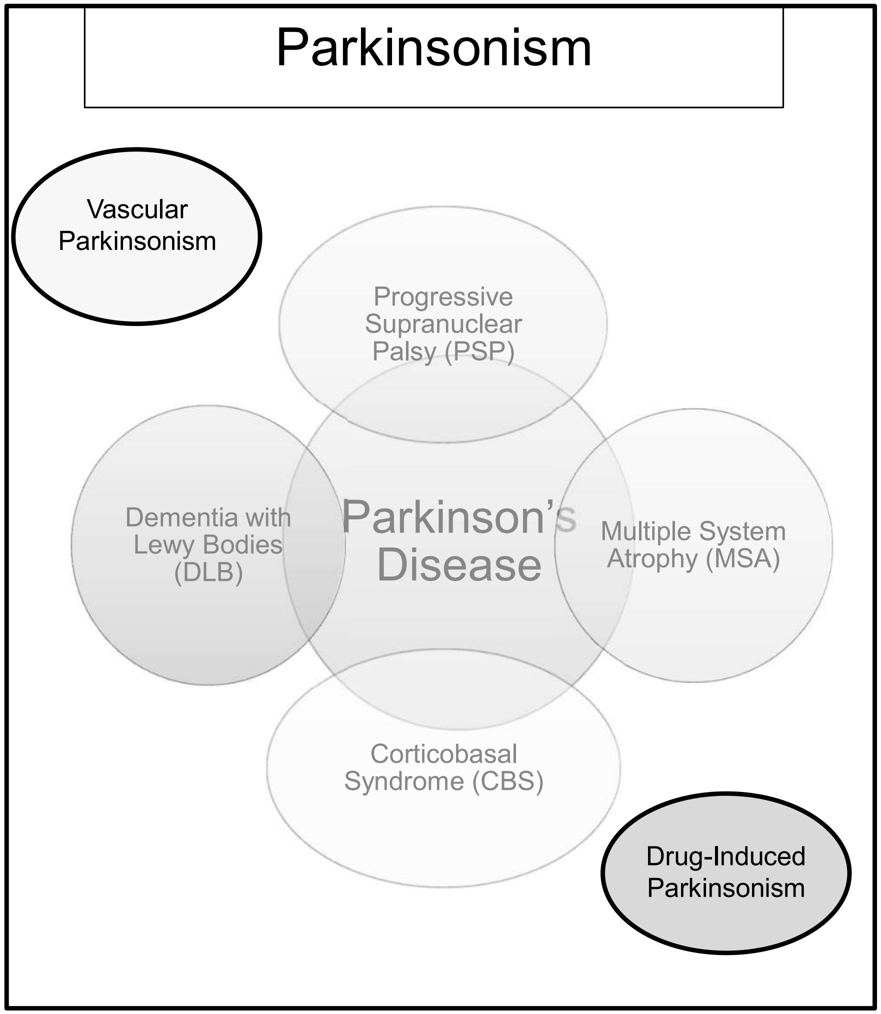
Parkinsonism
Vascular Parkinsonism
Progressive Supranuclear Palsy (PSP)
Dementia with Lewy Bodies (DLB)
Parkinson's Disease
Multiple System Atrophy (MSA)
Corticobasal Syndrome (CBS)
Drug-Induced Parkinsonism

Dementia with Lewy Bodies (DLB): Core Clinical Features

- Fluctuations in cognition and/or arousal
- Recurrent, fully formed visual hallucinations
- Spontaneous parkinsonism
- REM sleep behavior disorder

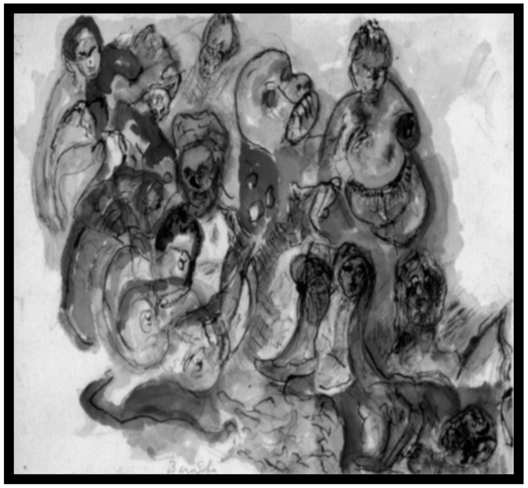

Frucht SJ, Bernsohn L. Visual hallucinations in PD. Neurology. 2002 Dec 24;59(12):1965.
McKeith IG, et al. Diagnosis and management of dementia with Lewy bodies: Fourth consensus report of the DLB Consortium. Neurology. 2017 Jul 4;89(1):88-100..

Lewy Body Dementia: Chicken & egg problem, under an umbrella

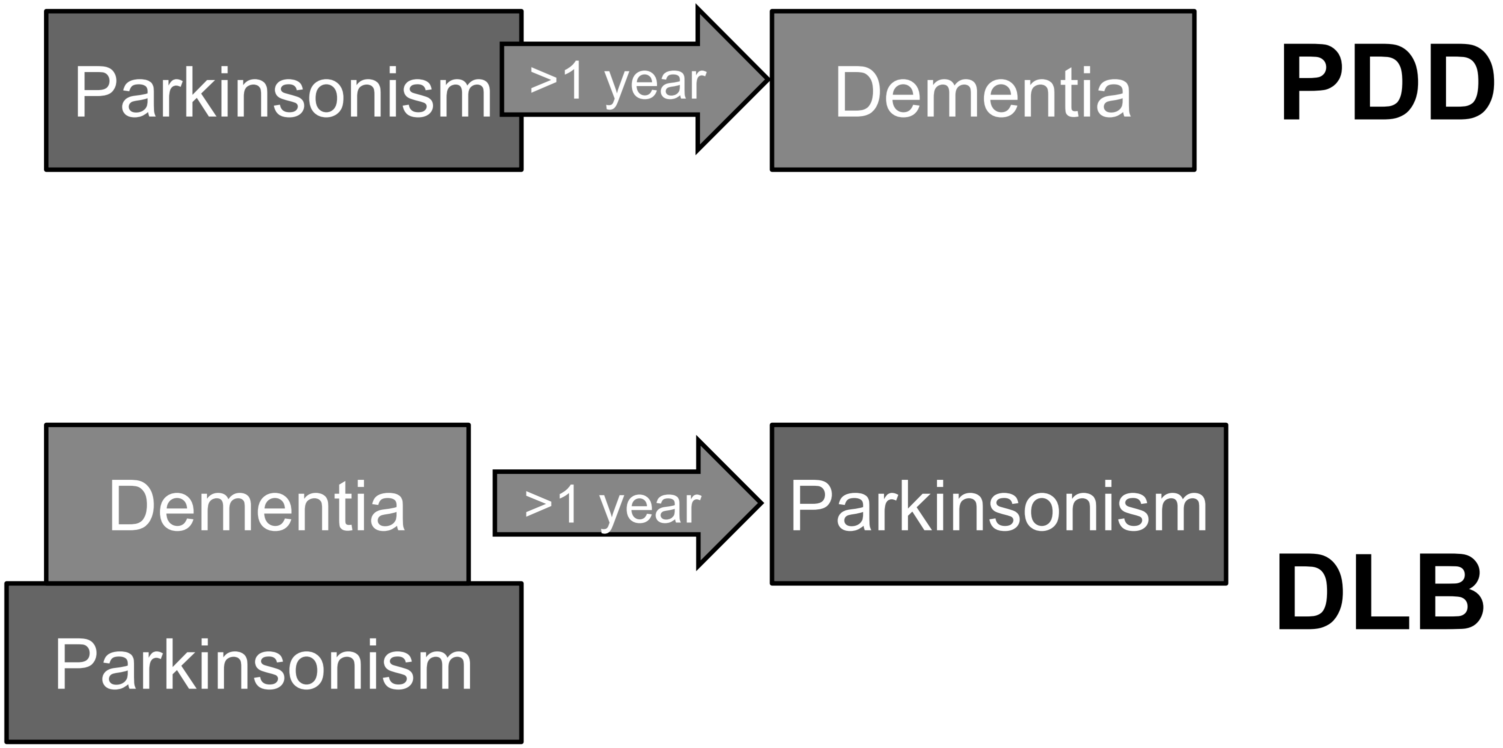

Multiple System Atrophy (MSA): Diagnostic Criteria

- Probable MSA:
  - Sporadic, progressive disease, onset after 30y, characterized by autonomic failure, including urinary incontinence (with erectile dysfunction in men), or an orthostatic decrease in BP by >30mmHg systolic or 15mmHg diastolic within 3 min of standing, plus $\geq$1 of:
    - Parkinsonism with poor response to levodopa (MSA-P)
    - Cerebellar syndrome: wide-based gait, uncoordinated limb movements, action tremor, nystagmus (MSA-C)

Fanciulli A, Wenning GK. N Engl J Med 2015;372:249-63.

MSA: Core Clinical Features

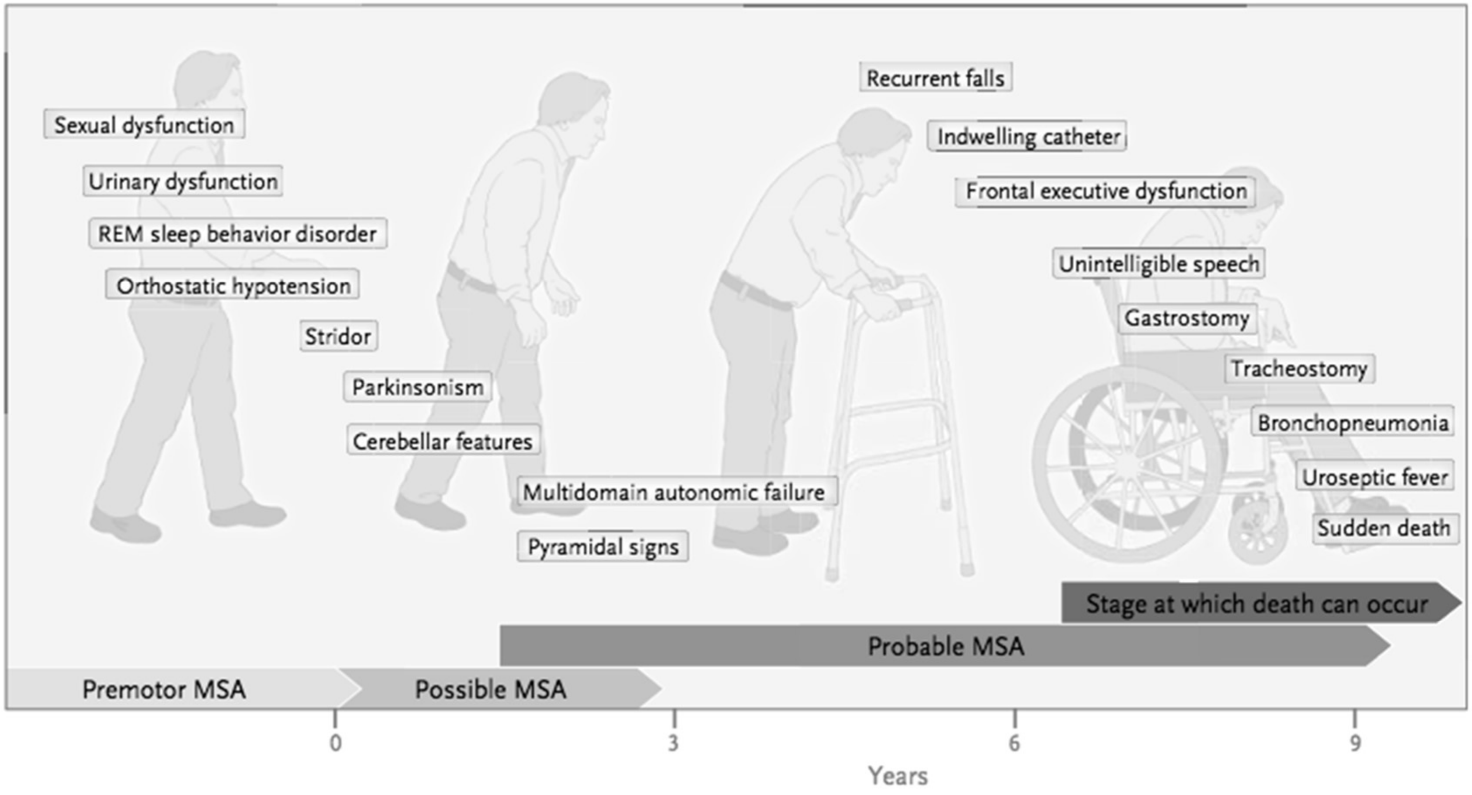

Figure 3. Natural History of MSA.
The premotor phase of MSA can last for months to years. Year 0 denotes the time of onset of motor symptoms. A diagnosis of definite MSA is not possible until the postmortem examination is performed.

Fanciulli A, Wenning GK. N Engl J Med 2015;372:249-63.

Case 3

Case 3 VIDEO

Progressive Supranuclear Palsy (PSP): Diagnostic Criteria

- Movement Disorder Society Criteria:
  - **Mandatory**: Sporadic occurrence; $\geq$40 yrs at onset; **and** gradual progression
  - **Core clinical features**:
    - **Ocular Motor Dysfunction:** Vertical supranuclear gaze palsy >> slow velocity of vertical saccades >> frequent square wave jerks *or* eyelid opening apraxia
    - **Postural Instability:** Repeated unprovoked falls within 3 yrs >> tendency to fall on pull test within 3 yrs >> more than 2 steps back on pull test within 3 yrs
    - **Akinesia**: Progressive gait freezing within 3 yrs >> parkinsonism, akinetic-rigid, predominantly axial, and levodopa-resistant >> parkinsonism, with tremor and/or asymmetric and/or levodopa responsive
    - **Cognitive dysfunction:** Speech/language disorder (nonfluent/agrammatic variant of PPA or progressive apraxia of speech); frontal cognitive/behavioral symptoms; corticobasal syndrome

Hoglinger GU, et al. Mov Disord. 2017 Jun;32(6):853-864.

PSP: Management

- Treatment is symptom specific:
  - Motor: carbidopa-levodopa, physical therapy
  - Dystonia: botulinum toxin injections
  - Mood: antidepressants
  - Vision: neuro-ophthalmology evaluation, prisms
- Recently failed trials
  - Riluzole, lithium, tideglusib (GSK-3 inhibitor), davunetide (microtubule stabilizer)
- Ongoing studies
  - Tau antibodies

curePSP
UNLOCKING THE SECRETS OF BRAIN DISEASE

Levin J, Kurz A, Arzberger T, Giese A, Höglinger GU: Dtsch Arztebl Int 2016; 113: 61–9.

Corticobasal Syndrome (CBS): Diagnostic Criteria

Table 4 **Proposed clinical phenotypes (syndromes) associated with the pathology of corticobasal degeneration[a]**

Syndrome	Features
**Probable corticobasal syndrome**	Asymmetric presentation of 2 of: a) limb rigidity or akinesia, b) limb dystonia, c) limb myoclonus plus 2 of: d) orobuccal or limb apraxia, e) cortical sensory deficit, f) alien limb phenomena (more than simple levitation)
**Possible corticobasal syndrome**	May be symmetric: 1 of: a) limb rigidity or akinesia, b) limb dystonia, c) limb myoclonus plus 1 of: d) orobuccal or limb apraxia, e) cortical sensory deficit, f) alien limb phenomena (more than simple levitation)
**Frontal behavioral-spatial syndrome**	Two of: a) executive dysfunction, b) behavioral or personality changes, c) visuospatial deficits
**Nonfluent/agrammatic variant of primary progressive aphasia**	Effortful, agrammatic speech plus at least one of: a) impaired grammar/sentence comprehension with relatively preserved single word comprehension, or b) groping, distorted speech production (apraxia of speech)
**Progressive supranuclear palsy syndrome**	Three of: a) axial or symmetric limb rigidity or akinesia, b) postural instability or falls, c) urinary incontinence, d) behavioral changes, e) supranuclear vertical gaze palsy or decreased velocity of vertical saccades

[a] See glossary of terms in appendix e-1 for further explanation of terms used.

Armstrong MJ, et al. Neurology. 2013 Jan 29; 80(5): 496–503.

Summary

- Bradykinesia + rigidity, tremor, or postural instability = parkinsonism
- Motor symptoms evolve over time, with fluctuations, freezing of gait, falls more common in advanced PD
- Levodopa doesn’t stop working…bandage multi-pack analogy
- Many non-motor symptoms are very treatable
- Hallucinations and dementia are common but not guaranteed
- Dementia does NOT mean depression and/or anxiety can’t coexist
- Sudden change = infection (UTI, URI, teeth, feet, seat, bowels) until proven otherwise
- Prominent dementia that precedes parkinsonism/motor symptoms: DLB
- Eye movement abnormalities, tendency to fall backwards, dysphagia: PSP
- Prominent orthostatic hypotension, urinary or bowel issues, ataxia: MSA

Questions? Onto Next Presentation!

Virtual Reality (VR) Experience

- **Introduction to "Dima Lab"**
  - 3 VR modules throughout the day
  - Each VR module will be introduced with a brief explanation of the scenario you are about to watch
  - Each scenario is broadcast through a headset and is having you "walk in the shoes" of a woman with Parkinson's
    - *Scenarios are approximately 10-15 minutes long*
    - *Write down any notes or questions you have*
    - *10-15 minutes after each scenario for discussion*

Break Time! See you in 10-15 Minutes!

Break-out Sessions

Break-out Sessions

What Nurses Need to Know
Serena Hess, RN MSN

Life with PD: Overview for PTs, OTs
Lauren Andersen, PT, DPT, NCS, CBIS
Kristie Trenkle, MOT, OTR/L, CLT

Life with PD: Overview for SLPs
Pat Brown, MA CCC/SLP

You will now be directed to your Zoom break-out room!

RUSH

Excellence is just the beginning.

Rush University Medical Center

Top Five Nursing Takeaways from Visits with Homebound PD Patients and Caregivers

March 5, 2021

Serena Hess, MSN, RN
Clinical Research Nurse

Let Me Introduce Myself

- Clinical Research Nurse for Dr. Fleisher for past 3 years
- Previously post-partum and then adolescent psych nurse
- Learned all the Parkinson's Disease (PD) terminology, medications, symptoms, etc. on the job!
- Coordinated and attended over 320 home visits with PD patients and their caregivers

Top Five Takeaways

- **Blood pressure measurements**
- **Management of “sudden” PD symptom changes**
- **Medication management and reconciliation**
- **Home safety**
- **Providing patients with tools and honest answers**

Blood Pressure Measurements

Orthostatic measurements

- Sitting blood pressure, both feet on floor, arm at heart level
- Standing blood pressure
  - *Ensure patient is stable (get help from caregiver if needed)*
  - *Stand for at least 1 minute (2-3 minutes preferable)*

Cuff Size

- If a patient is less than 100 lbs., **please look at his or her arm size.** Often times the smaller and frailer patients require a pediatric cuff (19.6-28.7 cm range)

Automatic vs Manual BP Reading

- If a patient has tremor or dyskinesia, the movement will not allow for a true automatic BP reading, so please do a manual reading

Blood Pressure Measurements

- As patients progress towards the end of their disease, their BP readings can vary throughout the day. Low blood pressure is **much** higher risk than occasional high blood pressure for PD patients.
- **High Blood Pressure Readings**
  - If they have **consistent readings over 170/100*** AND new symptoms listed below, alert the MD:
    - *Chest pain*
    - *Shortness of breath (SOB)*
    - *Abrupt vision changes*
    - *Severe headache*

.

Blood Pressure Measurements

- **Low Blood Pressure Readings**
  - If they have **consistent readings under 80/50*** AND new symptoms listed below, alert the MD:
    - *Loss of consciousness for over a minute*
    - *Fall leading to loss of consciousness or bleeding that does not stop within a few minutes*
    - *Diminished alertness or responsiveness*
    - *If there is one abnormal reading and the patient is dizzy, please elevate his legs, hydrate him, and retake BP after 10 minutes.*

Management of "Sudden" PD Symptom Changes

Patient or caregiver reports "sudden" changes (changes that occur within a 2-5 day timeframe; not gradual changes) in the below motor and/or non-motor symptoms

- Motor symptoms:
  - *Increased tremors or dyskinesias*
  - *Increased falls*
  - *Significantly slower or stiffer*
  - *New trouble transferring into/out of bed, chair, toilet*
- Non-motor symptoms
  - *NEW or increased hallucinations or delusions*
  - *Increased cognitive decline*
  - *Increased urination or constipation for 5+ days*

Management of “Sudden” PD Symptom Changes

<u>#1 “Go To” Move?</u>

- **Check for infection!**
  - **UTI (urinalysis and culture)**
  - **URI**
  - **Bowel obstruction or impacted stool**

Medication Management and Reconciliation

- **Medication Reconciliation:**
  - Comparing actual patient med list to bottles located in their house
  - Checking:
    - *Correct medication, dose (amount and frequency)*
    - *Expired bottles OR discontinued medications OR missing meds*
  - Seems basic, right?
    - *Because of the cognitive/motor challenges and caregiver strain that occurs in this population, med reconciliation can be tricky*

Medication Management and Reconciliation

Patient independence:

- VERY hard for many of our previously high-functioning patients to give up the control of medication management AND administration
- Cause for much strife between caregiver and patient and can lead to caregiver “giving up” and patient not getting the correct medications
- We saw this over and over again in our home visit patients and sometimes the patient just needs to hear from a professional individual (home health RN, PT, MD, etc.) that current med situation is NOT working and therefore the patient’s medications are NOT working

Medication Management and Reconciliation

- **Tips on how to help patient and caregiver:**
  - Calmly explain that if the patient cannot get the correct medications at the correct time, their symptoms will NOT improve
  - Discover the issue
    - *Divvy up tasks*
      - Caregiver orders medications and obtains them
      - Caregiver AND patient fill up pill box every Sunday morning
      - Caregiver AND patient set alarms for medication timing; if alarms do not work and patient STILL missing meds, then caregiver needs to administer
      - Patient has a daily med list that he can check off each med once he takes it

Home Safety

At the first home visit, RN would do a “safety assessment,” which included but was not limited to asking the following:

- How the patient can navigate stairs; how many stairs to enter and exit house; how many stairs within house?
- Does the patient have the following or need of the following:
  - Bathroom:
    - *Raised toilet seat, shower chair, grab bars in shower/bath or around bathroom*
  - Kitchen:
    - *Ample, non-spoiled food in fridge and pantry, safe cooking surfaces, fire extinguisher and fire alarms*
  - Bedroom:
    - *Unsecured/loose throw rugs, hard surfaces or corners near bed, need for grab bar on bed*
  - Other:
    - *Evidence of hoarding or severe clutter, pets that could trip them, slip or fall hazards at entrances and exits*

Providing Patients with Tools and Honest Answers

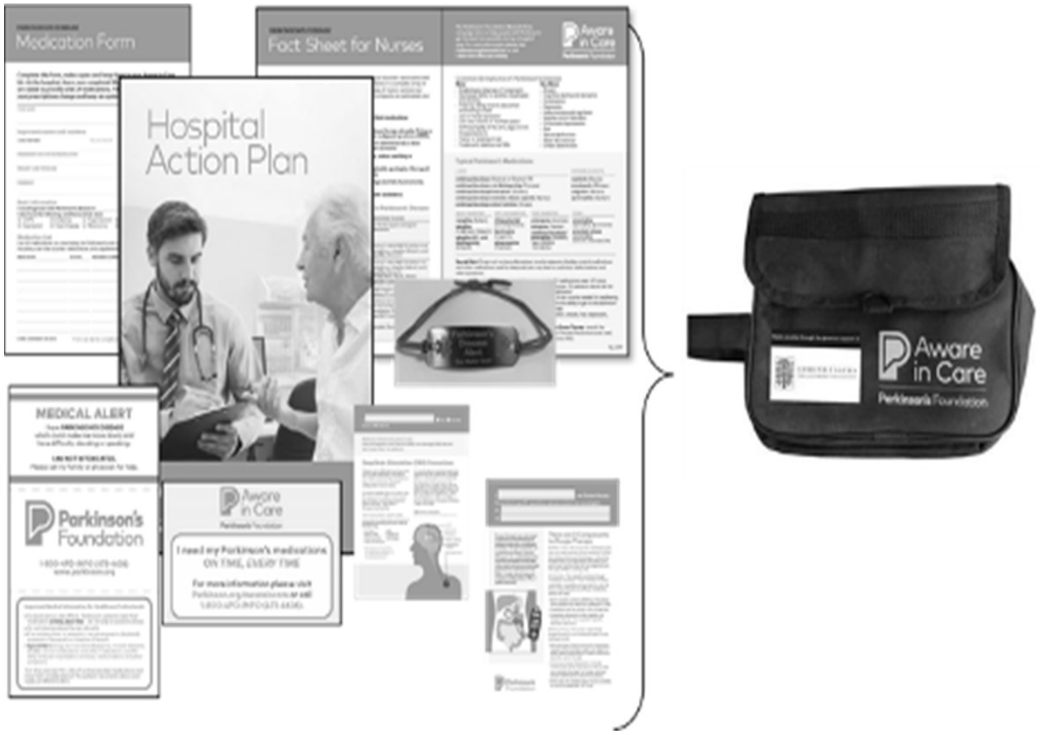

https://www.parkinson.org/Living-with-Parkinsons/Resources-and-Support/Hospital-Kit

Parkinson's Hospital (Aware in Care) Kit:

- Let's go through it!
- How to use it:
    - Bring it to home visits
    - Show patient how to order:
        - Parkinson's Foundation
        - Search "Parkinson's Hospital Kit"
        - Patient should bring the kit with them with any ER visit or planned hospitalization stay

Providing Patients with Tools and Honest Answers

Questions to possibly ask patient and caregiver:

- **Do you have an Advance Directive? Do you have a DNR?**
  - If yes, both should be in a place that is accessible during COVID times (binder or folder by the door with “In Case of Emergency” written on it)
  - If no, maybe see if patient and caregiver would like a social work consult either through your home health organization or through ordering provider

Questions YOU might be asked by patient and caregiver:

- **Do you believe my diagnosis? What is my prognosis?**
- **Do you feel it is time for us to look for a paid caregiver? Is it time for us to place my loved one in a “home?”**
- **When do we know it is time for hospice?**

Questions Before Patient Scenarios?

Scenarios Encountered in Patients' Homes

- **Scenario #1:**
  - 89-year-old homebound woman, PD diagnosis of 15 years, lives alone (paid caregiver ~ 5 hrs/day; family member caregiver at night) on oxygen, carbidopa-levodopa 25-100 mg 2 tabs QID, weighs approximately 92 pounds, intermittent tremors
    - *How would you take her blood pressure?*
    - *Manual or automatic cuff?*
    - *What kind of cuff?*
    - *BP readings are:*
      - 130/90, 77 (sitting); 115/85, 87 (standing).
      - Patient has no s/s of headache, impaired vision OR light-headedness, SOB. Would you call MD to report these values?

Scenarios Encountered in Patients' Homes

Scenario #2:

- 81-year-old man, PD diagnosis of 11 years, can leave home with wheelchair and caregiver, lives with wife (primary caregiver), some apathy and mild cognition issues, but complacent with medication regimen, fall risk primarily in bathroom, carbidopa-levodopa 25-100 mg, 2 tabs QID, donepezil 10 mg qhs. Over the weekend, wife notices that patient is falling more in the bathroom, has started "fighting her on taking medications," and cannot tell her what day it is. He is wetting the bed consistently every night. His tremors have gotten worse as well.
  - What would be your first step?
  - What searching questions would you ask the wife and patient?
  - Are there tests you would recommend for the PCP or ordering provider?

Scenarios Encountered in Patients' Homes

Scenario #3:

- 75-year old man, PD diagnosis of 20 years, homebound for the past year, lives with wife (primary caregiver), previously an engineer, depression and cognition issues, fall risk. Following medications:
  - Carbidopa-levodopa, 25-100 mg, 9 am (1.5 tabs), 11:30 am (1tab), 3 pm (1 tab), 6 pm (1.5 tabs)
  - Sertraline, 150 mg, qhs
  - Wellbutrin, 300 mg, qhs
- While completing medication reconciliation, you notice the carbidopa-levodopa bottle being used has an expired date and that the Wellbutrin is missing
- What would some of your next steps be?

Questions?

Thank you!
The work you do and
care you give these patients
means so much to them!

Life with PD: Overview for PTs and OTs

Presented by:

Lauren Andersen, PT, DPT, NCS, CBIS
Kristie Trenkle, OTR/L, CLT

All other planners, editors, faculty and reviewers of this activity have no relevant financial relationships to disclose. This presentation was created without any commercial support.

Learning Objectives

- Discuss the background of Parkinson’s Disease and benefits of multidisciplinary treatment
- Note the similarities and differences between PT and OT in setting of Parkinson’s Disease
- Motor and nonmotor deficits of Parkinson’s Disease
- Hoehn and Yahr Stages of Parkinson’s Diseases
- PT and OT treatment tips for each stage
- Outcome Measures

Lauren Andersen, PT, DPT, NCS, CBIS

Lauren earned her DPT from University of Illinois-Chicago in 2009 and has been practicing at Rush in both inpatient rehab and outpatient settings since. She achieved her Neurological Clinical Specialization in 2017 and became a Certified Brain Injury Specialist in 2019. She is currently the lead neurological physical therapist at Rush Physical Therapy, a division of Select Medical, where she participates in several multidisciplinary neurological based clinics. She is also currently studying part time towards achieving her Masters in Health Systems Management at Rush University.

Kristie Trenkle, OTR/L, CLT

Kristie Trenkle earned her Masters degree in Occupational Therapy in 2008 at Midwestern University. She has worked in both the inpatient and outpatient setting and treats a variety of patient diagnosis. She is specialized in lymphedema/oncology rehab. She also works in the driving rehab program at Rush and participates in research related to the benefits of providing therapy services to the Parkinson's population through tele-health.

Overview of Parkinson’s Disease

- Parkinson’s Disease is a complex neurological disorder with wide reaching implications for patients and their families
- Progressive in nature
- Patients face difficulty with ADLs and various aspects of mobility
- Leads to decreased independence, inactivity, social isolation and decreased quality of life

Management of Parkinson’s Disease

- **Traditionally centered on drug therapy**
  - Carbidopa-Levodopa as "gold standard" treatment
- **Even with optimal medical management, patients continue to deteriorate in function and participation**
- **Multidisciplinary management has been shown to be optimal for improving function for patients with Parkinson's Disease**

What is Physical Therapy?

- The use of treatments to develop, maintain, and restore normal body movement and physical function
- Can help clients recover from an injury, relieve pain, or deal with a chronic condition
- Goal of physical therapy is to improve health and quality of life

Role of Physical Therapy in Parkinson's Disease

- Fall prevention
- Wellness promotion
- Improve mobility
- Enhance cardiovascular fitness
- Postural re-education
- Improve quality of life

What is Occupational Therapy?

- The use of treatments to develop, recover, or maintain the daily living and work skills of people with a physical, mental, or developmental condition.
- A client-centered practice that places a premium on progress towards the client's goals.
- Focus on adapting the environment, modifying the task, teaching the skill, and educating the client/family in order to increase participation in and performance of daily activities, particularly those that are meaningful to the client.

Role of Occupational Therapy in Parkinson's Disease

- **Assisting patients maintain independence in daily activities, important life roles, and leisure activities (remediative vs restorative approach).**
- **Developing a home safety plan to reduce fall risk.**
- **Addressing cognitive issues that affect daily participation.**
- **Care giver Education.**
- **Mindfulness training.**

Motor Symptoms in Parkinson's Disease

- **Four primary motor symptoms – observing at least two is one way physicians diagnose Parkinson's**
  - Tremor
  - Rigidity
  - Bradykinesia
  - Postural instability

- **Other symptoms include gait difficulties, dystonia, and vocal symptoms**

Non Motor Symptoms of Parkinson's Disease

- Disturbances in sense of smell
- Sleep problems
- Depression and anxiety
- Pain
- Psychosis
- Fatigue
- GI issues
- Lightheadedness
- Urinary issues
- Sweating
- Personality changes
- Vision changes

Tremor

- Present at rest
- Usually disappears or decreases with movement
- Usually 4-7 beats / second
- May be cosmetically disabling, though usually does not interfere with ADLs in earlier stages

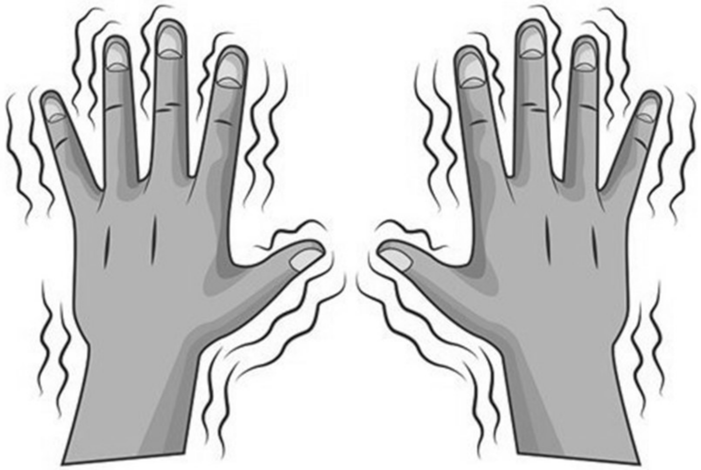

Rigidity

- Increased resistance to movement occurs
- The patient may perceive to be moving faster than they actually are
- Inability to adjust to equilibrium perturbations
- May increase energy expenditure, contributing to fatigue
- May be characterized as “lead pipe” or “cogwheel”

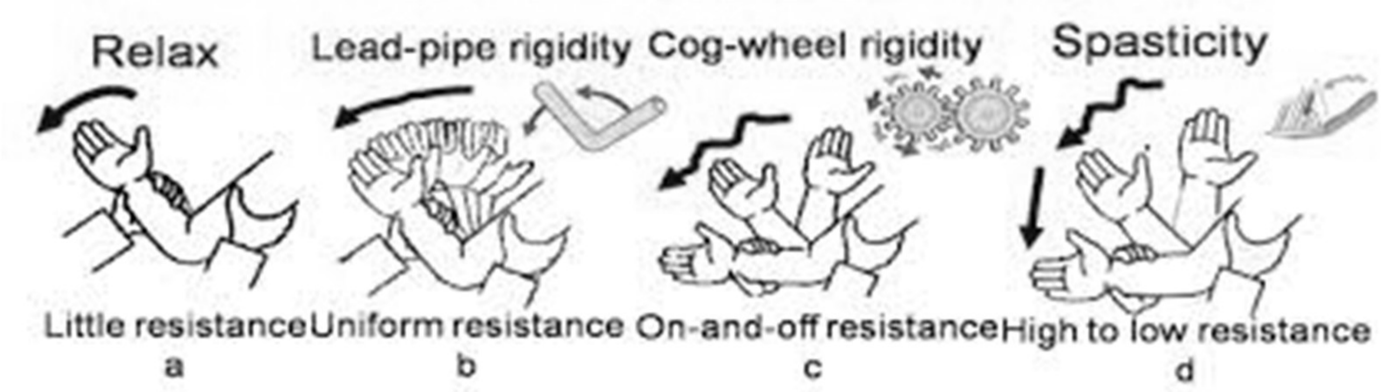

https://samarpanphysioclinic.com/spasticity-rigidity/

Bradykinesia / Akinesia

- **Bradykinesia = Decrease in motion**
- **Akinesia = Lack of motion**
- **Characterized by an inability to initiate and perform purposeful movements**
- **Complex, multi-joint movements more affected**
  - Decreased arm swing
  - Limited smiling at a funny story
- **Decreased activity in supplementary motor cortex, premotor cortex, and motor cortex**

Postural Instability

- Increased risk of falls and the sequelae of falls
- People with Parkinson's Disease are nine times are likely to fall compared to age matched control subjects

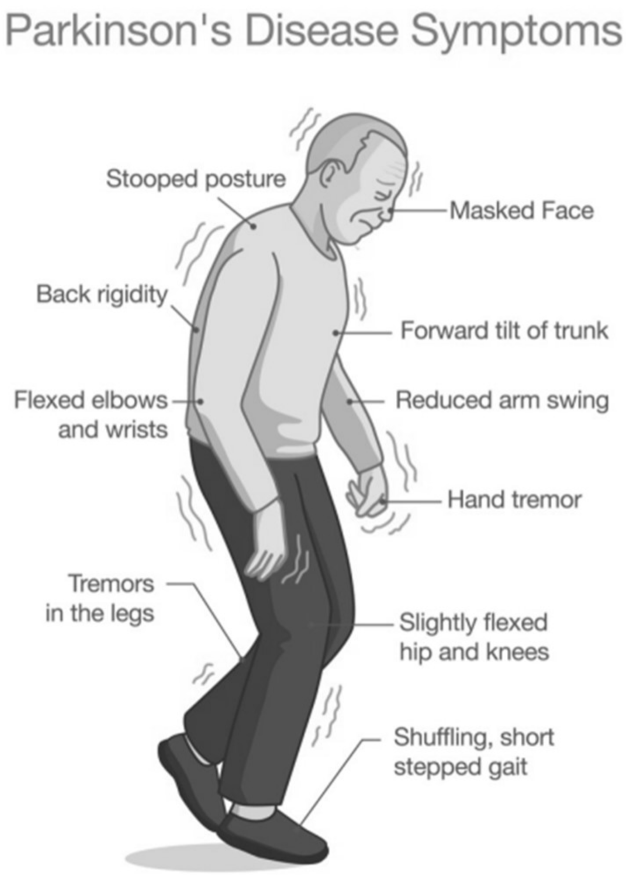

https://www.labiotech.eu/trends-news/axovant-parkinsons-disease-gene/

Gait Deficits

- **Decreased stride length / shuffling steps**
- **Decreased gait speed**
- **Festination**
  - Forward: propulsion
  - Backward: retropulsion
- **Freezing of gait**
- **Decreased arm swing**
- **Poor heel strike**

Figure 1: Cardinal symptoms of Parkinson's disease[4]

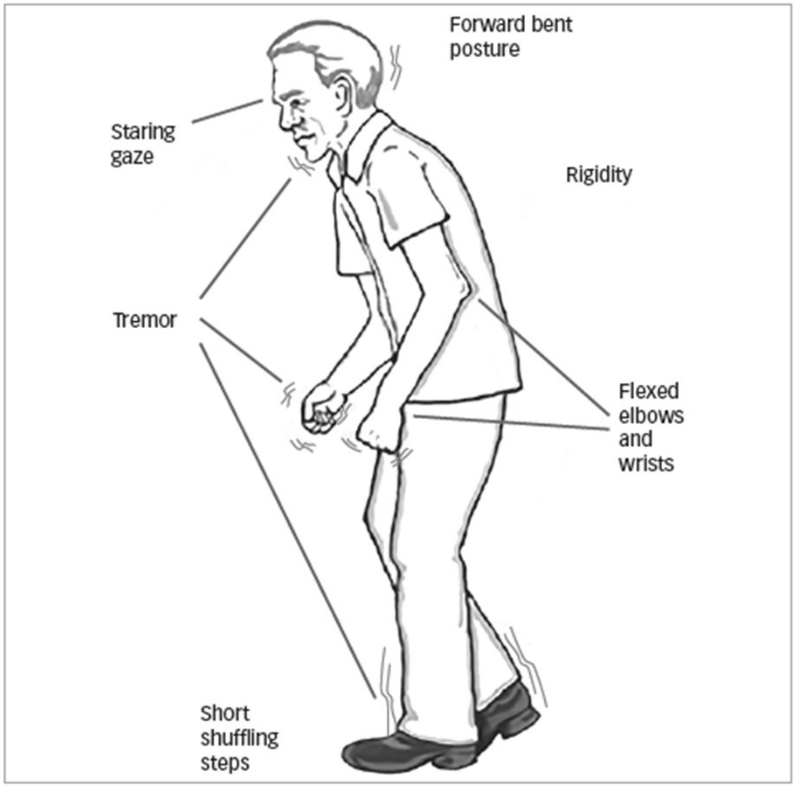

Adapted from information presented in Rodriguez-Oroz et al., 2009.[4]

Hoehn and Yahr Stages of Parkinson's Disease

Table 1: Comparison of the Original and Modified Hoehn and Yahr Scales

Hoehn and Yahr Scale	Modified Hoehn and Yahr Scale
1: Unilateral involvement only usually with minimal or no functional disability	1.0: Unilateral involvement only<br><br>1.5: Unilateral and axial involvement
2: Bilateral or midline involvement without impairment of balance	2.0: Bilateral involvement without impairment of balance<br><br>2.5: Mild bilateral disease with recovery on pull test
3: Bilateral disease: mild to moderate disability with impaired postural reflexes; physically independent	3.0: Mild to moderate bilateral disease; some postural instability; physically independent
4: Severely disabling disease; still able to walk or stand unassisted	4.0: Severe disability; still able to walk or stand unassisted
5: Confinement to bed or wheelchair unless aided	5.0: Wheelchair bound or bedridden unless aided

Goetz CG, et al. Mov Disord. 2004 Sep;19(9):1020-8.

Hoehn and Yahr Stage 1

- **Symptoms are mild**
- **Usually presents with tremor of 1 limb.**
- **Changes in posture, walking, and facial expression occur.**
- **Diagnosis may be missed**

Physical Therapy for Stage 1 PD

- **Exercise throughout a full range of motion is key in early stages to prevent muscular changes**
  - LSVT Big
- **Aerobic exercise**
  - Treadmill training, hiking, swimming, dancing (especially the tango)
- **Resistance training**
  - Push ups, lunges, resistance band exercises
- **Stretching**
  - Yoga, Pilates, daily stretching
- **Group classes**
  - Local YMCAs and support groups

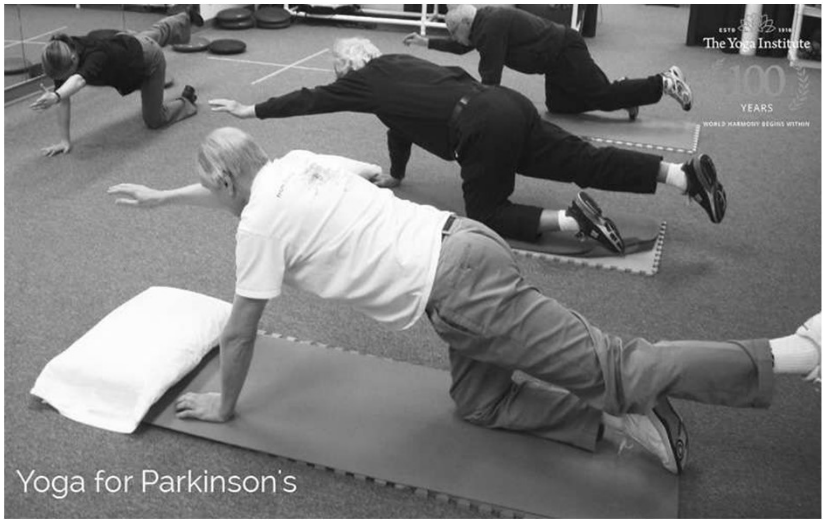

https://theyogainstitute.org/yoga-therapy-for-parkinsons/

Occupational Therapy for Stage 1 PD

- **Fine motor exercises to address deficits related to tremors in hand.**
- **Exercises that challenge motor planning to improve patient's ability to complete functional tasks.**
- **Adaptive strategies as needed.**

Outcome Measures for Stage 1 PD

- **Parkinson's Disease Questionnaire: a self report questionnaire which assesses Parkinson's disease-specific health related quality over the last month**
  - PDQ-39
  - PDQ-8 (short version)

Hoehn and Yahr Stage 2

- **Symptoms are bilateral.**
- **Posture/gait begin to be impacted**
- **Tremor, Rigidity**
- **Pt is able to live alone, but daily tasks are more difficult and take a longer time to complete.**
- **Daily activities and timing with medications.**
- **Managing fatigue.**

Physical Therapy for Stage 2 PD

- **Not using assistive device for gait**
- **Treatments similar to Stage 1**
  - Amplitude based
  - Multiplanar movement
  - LSVT Big
- **May begin to have stooped posture or neck / back pain due to rigidity**
  - Stretching through trunk and legs
  - Trunk extension based exercise

theparkinsonsfitnessproject

Occupational Therapy for Stage 2 PD

- Educate the patient to allow increased time as able to complete daily activities.
- Adaptive equipment for functional tasks that requires more challenging motor movements to complete (using built up handles, jar openers, elastic shoe laces, etc.).
- Toileting: regular schedule, avoid caffeinated drinks, as increased urinary frequency/urgency tends to occur at this stage.
- Home safety training.
- Cognitive screening/intervention

Hoehn and Yahr Stage 3

- **Significant slowing of body movements**
- **Early impairment of equilibrium while walking/standing, and during functional tasks.**
- **Falls are more common**
  - Inability to make rapid, automatic, and involuntary adjustments

Physical Therapy for Stage 3 PD

- **Consider assistive device for safety**
- **More pronounced festination and freezing, implement strategies into treatment**
    - Visual cues: stepping into agility ladder, over hurdles, over cones
    - Incorporate multiple planes of movement
    - Auditory cues: stepping to metronome, exercises to the beat of a song

Occupational Therapy for Stage 3 PD

- **Safety with functional transfers and ADLs.**
- **Ongoing home safety training.**
- **Safety when toileting in the middle of the night to reduce fall risk.**
- **Ongoing cognitive intervention.**

Hoehn and Yahr Stage 4

- **Rigidity and bradykinesia**
- **Tremor may be less than earlier stages.**
- **Symptoms are severe and limiting.**
- **Possible to stand without assist, but mobility likely requires walking device.**
- **The patient needs help with ADLs and is unable to live alone.**

Physical Therapy for Stage 4 PD

- **Patient likely needs assistive device for safety**
- **LSVT modifications in sitting and supine**
  - Research not validated
- **Increased shuffling, more consistent visual cues during gait:**
  - Laser cane / U Step walker
  - Thera band tied to front of walker
- **Increased freezing, especially during transitions**
  - Tape around door frame

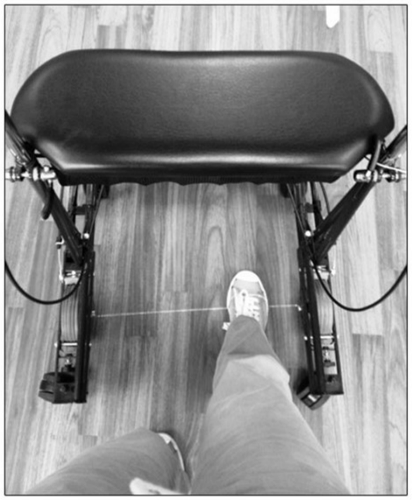

Ustep.com

Occupational Therapy for Stage 4 PD

- Increased difficulty with bathroom tasks, requiring more equipment recommendations.
- Care giver training.
- Bed mobility (hospital bed, bed rails, appropriate technique to get in and out of bed)

Outcome Measures for Stages 2-4 PD

- Mini BESTest
- Montreal Cognitive Assessment (MoCA)
- 5 times sit to stand test
- 6 minute walk test
- 9 hole peg test
- Functional Gait Assessment
- 10 meter walk test
- PDQ-39

Hoehn and Yahr Stage 5

- **Most advanced and debilitating stage.**
- **Patient requires wheelchair or is bed bound.**
- **Requires constant care.**
- **Patient may experience delusions and hallucinations.**

Physical Therapy for Stage 5 PD

- **Caregiver training for safety during functional mobility**
  - Sit to stand
  - Transfers
  - Assist for walking and ambulation
- **Ensure home is appropriate for wheelchair: appropriate inch turning radius**
- **Significant rigidity present: stretching education**

Occupational Therapy for Stage 5 PD

- Caregiver education: body mechanics, planning ahead, turning in bed, bathing/toilet hygiene in bed, transfer training (in/out of bed to chair, bathroom transfers, slide board).
- PROM education
- Lift equipment

Outcome Measures for Stage 5 PD

- Parkinson's Fatigue Scale

www.sralab.org/rehabilitation-measures

Parkinson’s Disease and the Ability to Drive

- As the patient progresses through stages of PD, their ability to drive will decrease.
- Many factors affected by PD will decrease their safety with driving: cognition, speed of processing information, motor control, reaction time, visual scanning, divided attention.
- Occupational therapy can assist the patient and family to be able to transition out of their role of driving.
- Rush outpatient Occupational Therapy Driving rehabilitation program.

Home Safety Training

- **Recommendation of appropriate DME.**
- **Home set up to promote ongoing independence and safety with mobility.**
- **Work simplification strategies.**
- **Managing fatigue throughout the day.**
- **Caregiver education.**

The Bigger Picture

- Parkinson's Disease is progressive and degenerative, cannot be halted or reversed
- Quality of life may be enhanced with combination of medication management, exercise, and therapy
- Combination of aerobic and strengthening exercises focusing on multiplanar movements and large amplitudes
- Increasing focus on safety throughout progression of disease
- Learning to use adaptive strategies to maximize independence through each stage.
- Caregiver support/training
- Home safety to decrease falls and increase independence with daily activities.

References

- American Parkinson Disease Association https://www.apdaparkinson.org
- Han, S. (2018). Understanding Parkinsonian Gait. *Healthline.* https://www.healthline.com/health/parkinsons/gait#symptoms
- Roeder, L, et al. (2013) Effects of Resistance Training on Measures of Muscular Strength in People with Parkinson's Disease: A Systematic Review and Meta-Analysis. *PLoS ONE.* 10(7)
- Shulman, L, et al. (2013) Randomized Clinical Trial of 3 Types of Physical Exercise for Patients With Parkinson Disease. *JAMA Neurology.* 70(2): 183-190
- Tomlinson, CL, et al. (2014) Physiotherapy for Parkinson's Disease: A Comparison of Techniques. *Cochrane Database of Systematic Reviews.*6(3)
- Umphred, D. (2007). Neurological Rehabiltiation: Fifth Edition. *Mosby Publishing.* pp. 783-793
- Wang, C. et al. (2015) Development of a human-like neurologic model to simulate the influences of diseases for neurologic examination training. *2013 IEEE International Conference on Robotics and Automation*, Karlsruhe, 2013, pp. 4826-4831
- Occupational Therapy Practice Framework: Domain & Process 2nd Edition (2008). American Journal of Occupational Therapy, 62, 625-683. doi:10.5014/ajot.62.6.625
- Foster et al. (2014). Systematic review of the effectiveness of occupational therapy-Related interventions for people with Parkinson's disease. The American Journal of Occupational T herapy, 68 (1), 39-45.
- Rao, A. (2014). Occupational Therapy in chronic progressive disorders: Enhancing function and modifying disease, 68 (3), 251-252

Thank You

Life with PD: Overview for SLPs

Pat Brown MA CCC/SLP

What SLPs Address with PD Patients:

- Voice
- Swallowing
- Communication/Cognition

For evidenced based practices, visit
https://www.asha.org/research/ebp/evidence-based-practice-catalog/

Concepts

-Each area addressed has concepts that the SLP addresses directly or indirectly.

-These concepts need repetition as many patients and their families or caregivers requires this repetition to process the concept and the behaviors associated with each concept.

-Embracing these concepts and the behaviors associated with them is dependent on how the patient accepts that they have a progressive degenerative disease.

Voice Concepts

- **The patient will have more problems with voice as their disease progresses.**
- **The patient's voice is also an indication of how well the patient is swallowing.**
- **The patient's limited mobility will limit his/her communication opportunities and the patient/caregivers may have to manufacture opportunities for the patient to speak aloud.**
- **The patient will have to do something daily to maintain his/her voice, either intensity or quality.**
- **The patient's ability to communicate will assist him/her to control their environment.**

Voice Strategies

Strategy	Pros	Cons	Considerations
LSVT	This is a systematic program to remediate and maintain vocal intensity. Patients/caregivers are able to practice exercises independently.	-With PDGM, therapy is often once a week, not 4 x week as required by the program.<br>-It is a program that is dependent to some extent on the patient being able to read printed words.	-Patient requires assessment of visual processing, reading, and cognition.
Sound Level Meters	-Easily obtained: available for free on most app stores<br>-Allows an objective measure of how loud the patient is	-Sound levels are presented quickly and may be difficult to read<br>-Patient and caregivers may require training to use this technology.	-Patient needs to have available devices: smartphones, computers or tablets.

Strategy	Pros	Cons	Considerations
Laryngeal Resistance Exercises:<br>-Shaker Exercises<br>-Modified Shaker Exercises<br>-Thera band Exercises	Assists the patient is maintaining a clear vocal quality and staving off the ‘Parkinson’s Gurgle’.	The patient may not have the physical capability to participate in these exercises.	PT/OT can be referred to assess the patient’s ability to physically participate in these exercises.
Breath Control Exercises:<br>-Whistles<br>-EMST/IMST devices	Use of EMST and IMST devices allow strengthening of respiratory musculature and to some extent, body core musculature.	Patient may have difficulty using EMST/IMST devices.	Patient dentition may prevent use of these devices.

Swallowing Concepts

- **The patient eats when it is the optimal time for him/her to eat.**
- **The patient eats 3 small meals and 3 small snacks.**
- **The patient has to maximize calories and minimize effort.**
- **The patient may safely consume multiple diet textures, even though the majority of what he/she eats may be one diet texture.**

Swallowing

Strategy	Pros	Cons	Considerations
Chin Tuck	-This technique is easy for the patient to use and may provide patient with adequate airway protection.<br>-This technique also may allow the patient to attend to the task at hand.	-The patient may aspirate bolus being swallowed.	Safety of use should be confirmed by MBSS.
Supraglottic Swallow (Breath Hold)	-This technique is easy for the patient to use and may provide adequate airway protection.	-The patient may not be able to 'hold' his/her breath, especially if the patient chews food excessively or has difficulty transferring a bolus anteriorly to posteriorly orally.	-Patient may need breath control exercises prior to use of breath hold.

Swallowing

Strategy	Pros	Cons	Considerations
Effortful Swallow	-This technique may assist patient to cope with difficulty initiating muscle movement to swallow quickly.	-Oral motor weakness and oral desensitization may hinder patient from easily propelling a bolus posteriorly.	-Patient may need cuing to perform this maneuver and someone to cue the patient will have to be available during the patient's meal.
Use of Straws	-The patient may be able to direct the flow the bolus in the oral cavity, especially if there is oral desensitization.<br>-The patient was able to drink liquids quicker and hence, drink more.<br>-Patient can drink thickened liquids via straw.	-The patient may obtain a bolus that is too large to manage orally and may aspirate liquids.	-Patient may need safety of use verified by MBSS.<br>-Disposable straws may become more difficulty to obtain and permanent use straws may be difficulty to sanitize.

Swallowing

Strategy	Pros	Cons	Considerations
Laryngeal Resistance Exercises:<br>-Shaker Exercises<br>-Modified Shaker Exercises<br>-Thera band Exercises	-This technique assists the patient in being able to clear pharyngeal stasis of residuals of the boluses of solids and liquids.	-This technique may not be effective in final stages of Parkinson's Disease.<br>-The patient may not be able to physically capable to participate in these exercises.	-The patient may require additional exercises to improve breath control and cough strength in conjunction with these exercises to be effective.<br>-PT/OT can be referred to assess the patient's ability to physically participate in these exercises.
Breath Control Exercises:<br>-Whistles<br>-PEP devices<br>-EMST/IMST devices	-These devices allow the patient to exhibit improved breath control for swallowing and reduce choking/aspiration risk.<br>-Improved breath control allows the patient to produce a stronger cough, improving airway protection.	-Patient may be unable to use these devices to achieve improved breath control in the later stages of the disease.	-The devices require sanitization frequently, especially if the patient has excessive saliva or residual from solids/liquids.

Device	Pros	Cons	Considerations
Sip A Mug	-Cheap!<br>-When the top is screwed on, liquids go up into the straw.<br>-May be used with thickened liquids.	-Facilities do not like use of these.<br>-Not dishwasher safe.<br>-Difficult to sanitize.<br>-It is clear and patients with visual processing deficits may have difficulty seeing it.	This drinking device is easily available and affordable for the patient and his/her family.
Pillow fort Milk Bottle	-Inexpensive.<br>-Dishwasher safe.<br>-Straw is attached to lid.<br>-May be used with thickened liquids.<br>-Holds 8 oz. of liquid.	-Patient needs to have a strong suck.<br>-Liquids do not stay up in the straw.	Facilities like this device as it is easy to clean.

Strategy	Pros	Cons	Considerations
Limited Sip Straw	-Limited bolus size of liquid to 6.2 ml.<br>-This device helps to train patient as to sensation of limited size bolus.	-Needs disposable straw.<br>-Straw needs to be cut in length to fit the device.<br>-Difficult to sanitize.<br>-Facilities tend to lose the device.<br>-Costlier.	-The patient may have increased difficulty with use with thickened liquids.<br>-Safety of thin liquid boluses may need to be verified by MBSS.
Limited Sip Cup	-Limited bolus size.<br>-Easy to use with thickened liquids.<br>-Dishwasher safe.	-Difficult for patient to use when he/she has fine motor deficits.<br>-Patient with visual processing deficits may have difficulty lifting the device to drink from it.	-Safety of thin liquid boluses may need to be verified by MBSS.

Device	Pros	Cons	Considerations
Nosey Cup	-Fits over patient's nose.<br>-Allows the patient to tip device up to obtain liquid bolus.<br>-Easily used with thickened liquids.<br>-Dishwasher safe.	-Hard for the patient to control the amount of the bolus.<br>-Patient with visual processing deficits may have a hard time tilting the device upward.<br>-Cheaper alternatives.	
Drink EZ Bottle	-This device electronically delivers a bolus via a straw with the push of a button.<br>-Limited bolus size.<br>-May be used with thickened liquids.	-Expensive.<br>-Difficult to use if patient has fine motor weakness.	-If possible, patient should be trialed with this device prior to purchase.<br>-Patient's safety with thin liquids may need to be verified by MBSS.

Supplement	Pros	Cons	Considerations
Boost Very High Calorie	-530 calories per 8 oz.<br>-Readily available<br>-High calorie, high protein with only 4 gm of sugar	-Only available in vanilla	
Benecalorie	-330 calories in 1/3 cup of soft pureed texture	-Can only be used easily in warm foods<br>-pricey	
Scandishake	-580 calories per 3 oz powder packet<br>-has many flavors	-expensive<br>-has 28 gm sugars	

Calorie Supplements

Supplement	Pros	Cons	Considerations
Ensure Plus	-350 Calories/8 oz.<br>-multiple flavors<br>-easily obtainable	-store brands may be cheaper<br>-22 gm sugars	
Carnation Instant Breakfast	-220 Calories without milk<br>-Has a sugar free version<br>-multiple flavors<br>-inexpensive<br>-has recipes on their website<br>-easily obtainable	-store brands may be cheaper<br>-need to mix with milk or some other liquid	
Protein Powder	-multiple brands of various pricing<br>-easy to use<br>-may appropriate for those who do not or cannot eat meat or secondary to chewing or dental problems<br>-easily obtainable	-need to mix with food or liquids	

Hydration

Patient may have difficulty maintaining hydration and may benefit from an electrolyte replacement:

- Zip Fizz
- Gatorade/G2 with Protein/G2 (Sugar free)
- Powerade
- Mio Sport
- Propel
- Electrolyte powders/tablets

Communication/Cognitive Concepts

- **The patient will have to engage in daily activities to maintain communication and cognitive skills.**

- **The patient will be easier to care for if her/his cognitive skills are maintained.**

- **The patient will be able to control his/her environment if communication skills are maintained**

Communication/Cognitive Skills

Most Frequent Areas Treated By SLPs In Home Health:

- Word Retrieval
- Safety Awareness
- Visual Processing
- Attentional Processing
- Memory

Word Retrieval

- **Evidence based practices**
- **Multiperson verbal games, such as:**
  - Scategories, Anomia, Trivial Pursuit, etc.
- **Reading aloud**
- **Support groups and activities**
- **Video chats/events**

Safety Awareness

- Fall Calendars
- Hydration Calendars
- Sit/Stand Procedures
- Movement Procedure/Precaution Books
- Family and Caregiver Training
- Role Playing
- Error Recognition

Visual Processing

- **Identification of Visual Processing Deficits**
- **Patient/Family/Caregiver Education**
- **Worksheets Addressing Visual Processing**
- **Reading Large Print**
- **Changing Print/Background Colors**
- **Mazes, Jigsaw Puzzles, Word Searches, Coloring, ID Differences Puzzles, etc.**
- **Visual Games, such as Heads Up, Win Lose or Draw, Pictionary, etc.**
- **Photography**

Attentional Processing

- Patient may have mild to moderate memory deficits, which appear worse secondary to attentional processing deficits.
- Patient/Family/Caregiver Education
- Computer Games
- Simple, Repetitive Tasks
- High Interest Activities
- Physical Endurance

Memory

- Evidence based practices
- Patient/Family/Caregiver Education
- Computer games such as Memorado, Memory Games for Adults, Left vs. Right, Sharply, etc.
- Board Games such as Senior Moments, Memory Maze, Trivial Pursuit, etc.
- Memorizing specific items, such as poetry, religious texts, quotations, jokes, etc.
- Compensatory memory techniques/strategies
- Use of technology

The End

VR Experience Part 2: Continuation of Dima Lab

Break Time! See you in 10-15 Minutes!

Voice of the Patient & Caregiver
Let's Welcome our Interactive Panel!

- **Moderator, Nurse Serena**
  - J., past spousal caregiver
  - C., past adult child caregiver
  - M. and B., PD patient and spousal caregiver (respectively)
  - M.J. and C., PD patient and formal caregiver (respectively)
- **Panel Goals**
  - Hear from our panel
    - *What worked/did not work with HHA professionals*
    - *What they wish their HHA professionals knew PRIOR to entering their homes*
    - *Questions from the participants*

VR Experience Part 3: Conclusion of Dima Lab

Break Time! See you in 10-15 Minutes!

RUSH

Excellence is just the beginning.

Rush University Medical Center

Detecting Urgent Situations in the Home: What to Look For

March 5, 2021

Jori E. Fleisher, MD, MSCE
Serena Hess, RN, MSN

Common Issues Seen During Home Visits

- Falls
- Mental status changes
- Concerns about neglect or abuse
- Caregiver frustration

Falls

- Common Fall Reasons
  - Orthostatic hypotension-related
  - throw rugs/poorly lit hallways/clutter-related
  - patient not compliant with walker or wheelchair
  - transfers

Mental Status Changes

Due to:

- infection
- sun downing/excessive napping during the day
- progression of the disease
- medication mismanagement OR need for medication

Suicidal Ideation (SI) and PD

- Unfortunately occurs within this chronic population
- Please use your agency's SI protocol and never hesitate to call 911
- If patient is "vague" about SI, always confirm that the most important task is to TELL SOMEONE (loved one, caregiver, hotline) if you have SI

Concerns about Neglect and Abuse

Signs:

- **Absence of food in the fridge and/or pantry**
- **Absence of medications that have been repeatedly ordered/never picked up**
- **Excessive clutter, vermin, or rotten food within house**
- **Excessive OR absent heating/cooling for the season**
- **Strong urine or fecal odors**

Concerns about Neglect and Abuse

How to Handle this

- **Absence of food in the fridge and/or pantry**
- **Excessive clutter, vermin, or rotten food within house**
- **Excessive OR absent heating/cooling for the season**
    - Is it hard to keep fresh food in your fridge or pantry? Do you know why?
    - Is it hard to keep your home clean? Have you reached out to your county or township for help?
    - Did you notice it is VERY hot and/or VERY cold in here? Does it bother you?
    - Social work consult

Concerns about Neglect and Abuse

- **Absence of medications that have been repeatedly ordered/never picked up**
  - It looks like you are missing some medications. Do you know why?
  - Mail order, medication “pack” companies, asking if it is need-based and reaching out to prescribing doctor’s team for medication tier reductions
- **Strong urine or fecal odors**
  - Are you bathing every day or at least every other day? If not, why?
  - If hard for caregiver, order CNA help, hopefully 2x’s/week
  - If patient is apathetic, explain that not bathing enough can lead to UTIs

If you feel there is possible neglect or abuse, please get a social work consult or bring in a colleague to make a plan

Hospitalizations

Times when patient SHOULD be sent to the hospital:

- falls with injury, especially head injury
- increased DANGEROUS delusions and VH in absence of infection
- patient is unconscious or repeatedly falling due to OH

Hospitalizations

Times when patient SHOULD NOT be sent to the hospital:

- repeated elevated asymptomatic BP
- one elevated or low asymptomatic BP measurement, that could be due to wrong cuff size, use of automatic cuff, increased dyskinesias or tremors

Tools to Have on Hand for Emergencies

Review:

- Bring Aware in Care kits to homes
- Show patients how to use and order them
- Explain how having at least these items on hand at all times will be beneficial for their care:
  - Emergency contact name and number
  - Contact name and number for:
    - *PCP*
    - *Movement Disorders Specialist*
  - List of medications with dosages and timing
  - Advance directives and/or DNR
- Have this all in a laminated folder or binder, close to the door

Q & A with Speaker Panel

- **Moderator, Jori Fleisher, MD MSCE**
  - Serena Hess, RN, MSN
  - Lauren Andersen, PT, DPT, NCS, CBIS
  - Kristie Trenkle, MOT, OTR/L, CLT
  - Pat Brown, MA CCC/SLP
- **Panel Goals**
  - Open Q & A from our participants

RUSH

Excellence is just the beginning.

Rush University

Wrap Up

Learning Objectives

- Discuss Parkinson's Disease and evidence-based management to provide in-depth coverage of advanced motor and non-motor symptoms for Parkinson's Disease.
- Recall how to recognize fall prevention and identify and perform home safety assessments to promote healthy aging-in place in Parkinson's Disease.
- Outline the importance of medication timing and reconciliation, signs and symptoms of orthostatic hypotension and constipation, and other specific challenges for homebound Parkinson's Disease patients.

Advanced Parkinson's Disease & Healthcare

- **Parkinson's Disease (PD) affects 1-2% of people $\geq$60 years old**
  - Variety of motor and non-motor symptoms, variety of presentations
  - Complex medication regimens
  - Uncoordinated care & heavy reliance on family caregivers
  - High rates of emergency room visits, hospitalizations, and nursing home placement due to **falls, neuropsychiatric symptoms,** and **infections… many of which are preventable if detected and managed early**

Parkinsonism vs. Parkinson's Disease

<u>Parkinsonism?</u>

- Bradykinesia
  (slow, small movements)

+ $\geq$1 of:

1. Rigidity
   (muscle stiffness)
2. Tremor
   (usually at rest and starting on one side)
3. Imbalance/posture changes

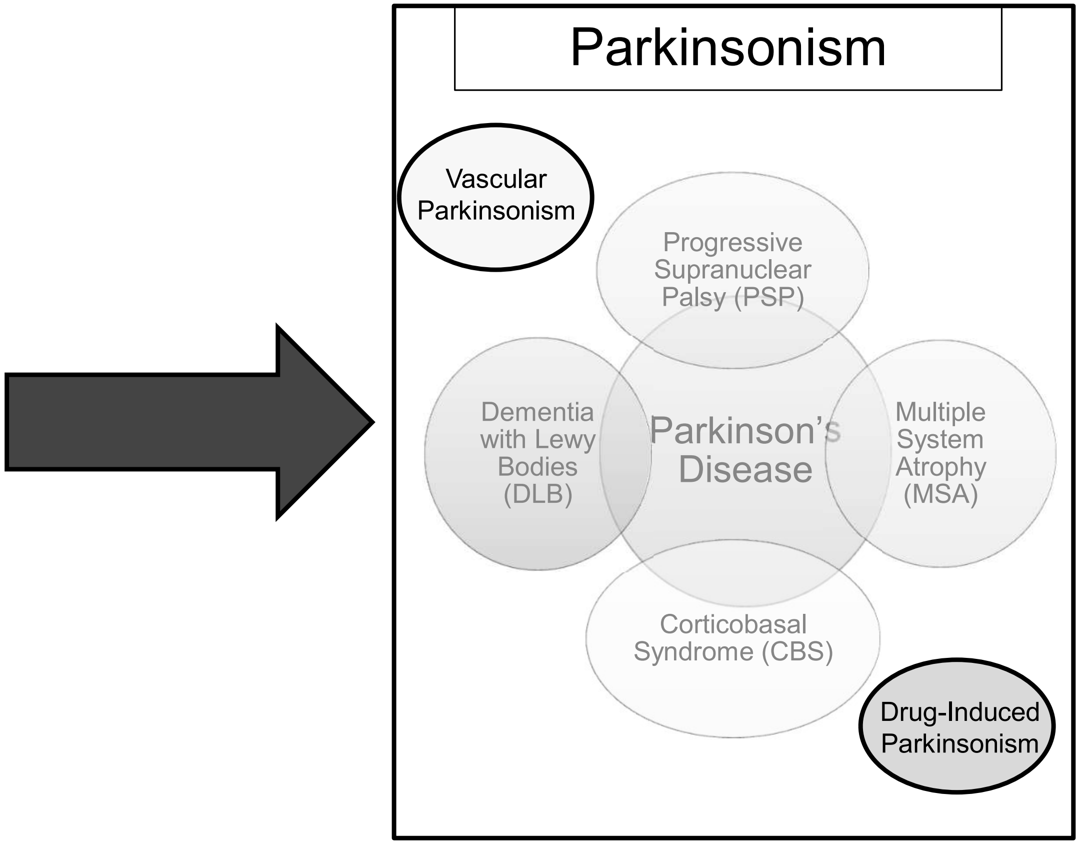

Evolution of Fluctuations

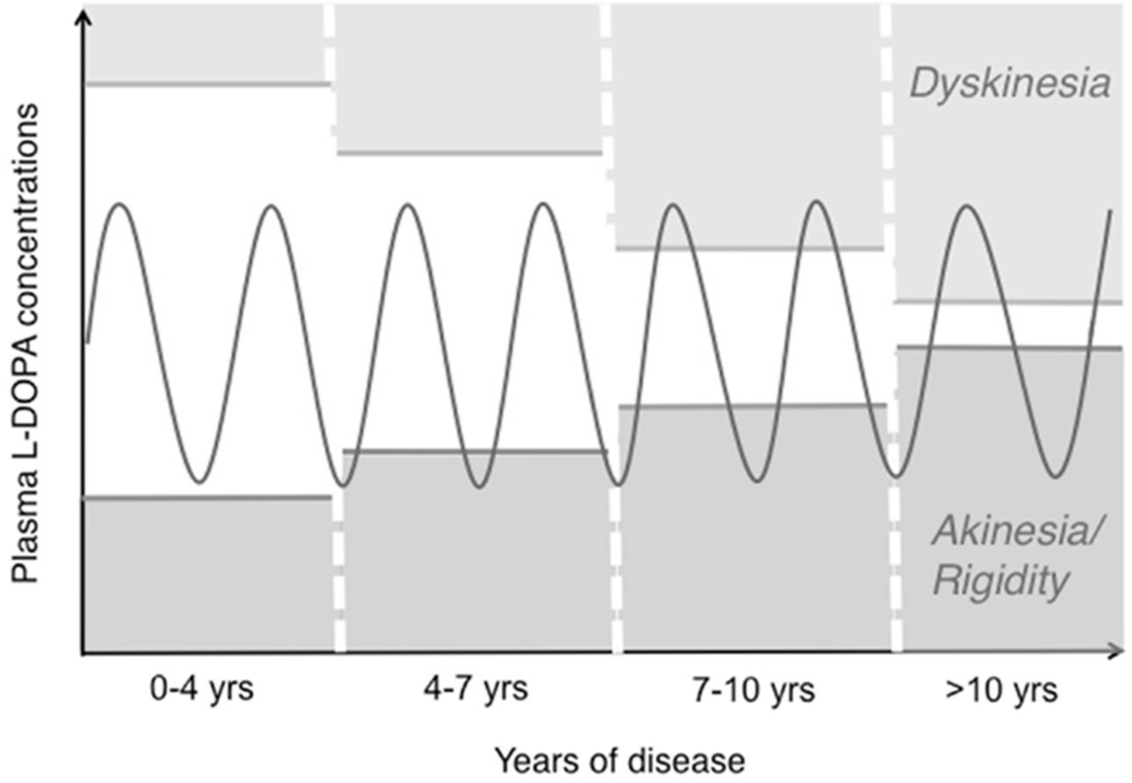

Cenci MA. Presynaptic Mechanisms of l-DOPA-Induced Dyskinesia: The Findings, the Debate, and the Therapeutic Implications. Front Neurol. 2014 Dec 15;5:242.

Falls

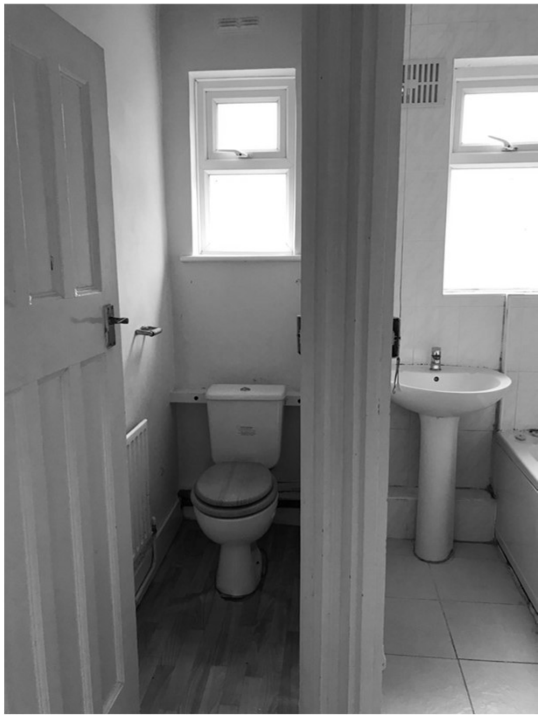

- Identify contributing factors
  - Wearing-off
  - Freezing of gait
  - Imbalance
  - Impulsivity
  - Orthostatic hypotension
- Identify setting
  - Absence of or inappropriate assistive device
  - Tight spaces—bathrooms, kitchen
  - Multitasking, particularly on stairs
  - Poor home safety
- Plan: Physical therapy & home safety evaluation

Depression

- **#1 predictor of quality of life in PD**
- 30-40% cumulative prevalence
  - *NOT* a reaction to the diagnosis
  - Related to serotonergic & noradrenergic changes
- Non-medication treatments:
  - Social involvement, support groups
  - Exercise
  - Cognitive-behavioral therapy
- Anxiety in up to 40%
- Dementia does not mean depression or anxiety go away

Barone P, et al. *Mov Disord*. 2009;24(11):1641-1649. doi:10.1002/mds.22643

Psychosis and Hallucinations

- Up to 60% prevalence
- Hallucinations:
  - **Illusion**: mistaking real object for something else
  - **Sensory illusions**: ‘passage’ hallucination, sensing a presence
  - **Visual hallucinations**: evolve from simple, non-threatening, preserved insight to complex, frightening, constant, limited insight
- Delusions:
  - **Paranoia**: generalized or specific; delusional jealousy
  - **Reduplicative paramnesias**: Capgras syndrome and others
    - ***Ask and you shall receive…***

Cannas A, et al. Neurol Sci. 2017;38(2):225-231.

Treatment Approach

- Simplify medication regimen to balance motor and cognitive difficulties
- Maintain a structured, familiar environment
- Maintain a regular sleep-wake cycle & circadian rhythm
- Individualized daily schedule
- Caregiver and family education & support

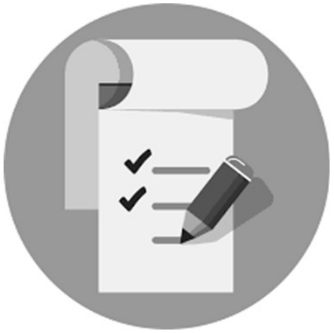

Blood Pressure Measurements

Orthostatic measurements

- Sitting blood pressure, both feet on floor, arm at heart level
- Standing blood pressure
  - *Insure patient is stable (get help from caregiver if needed)*
  - *Stand for at least 1 minute (2-3 minutes preferable)*

Cuff Size

- If a patient is less than 100 lbs., **please look at his or her arm size.** Smaller and frailer patients may require a pediatric cuff (19.6-28.7 cm range)

Automatic vs Manual BP Reading

- If a patient has tremor or dyskinesia, the movement will not allow for a true automatic BP reading, so please do a manual reading

Blood Pressure Measurements

- As patients progress towards the end of their disease, their BP readings can vary throughout the day. Low blood pressure is **much** higher risk than occasional high blood pressure for PD patients.
- **High Blood Pressure Readings**
  - If they have **consistent readings over 170/100*** AND new symptoms listed below, alert the MD:
    - *Chest pain*
    - *Shortness of breath (SOB)*
    - *Abrupt vision changes*
    - *Severe headache*

Blood Pressure Measurements

- **Low Blood Pressure Readings**
  - If they have **consistent readings under 80/50*** AND new symptoms listed below, alert the MD:
    - *Loss of consciousness for over a minute*
    - *Fall leading to loss of consciousness or bleeding that does not stop within a few minutes*
    - *Diminished alertness or responsiveness*
    - *If there is one abnormal reading and the patient is dizzy, please elevate his legs, hydrate him, and retake BP after 10 minutes.*

Medication Management and Reconciliation

- **Medication Reconciliation:**
  - Comparing actual patient med list to bottles located in their house
  - Checking:
    - *Correct medication, dose (amount and frequency)*
    - *Expired bottles OR discontinued medications OR missing meds*
  - Seems basic, right?
    - *Because of the cognitive/motor challenges and caregiver strain that occurs in this population, med reconciliation can be tricky*

Sudden Changes & Culprits

- Abrupt changes in mobility – diminished efficacy of medications, increased OFF time, increased freezing of gait
- Abrupt changes in cognitive status
- New or significantly worsened hallucinations or delusions

- Exclude reversible causes
  - **UTI, UTI, UTI**
  - **COVID,** URI, (aspiration) pneumonia
  - Other infectious causes (teeth, seat, feet)
  - Impaction or bowel obstruction
  - Metabolic derangements, dehydration
  - Medication errors or new medications

Don’t wait!

- **By the time patients develop “typical” signs and symptoms, it may be too late**

Home Safety

- How the patient can navigate stairs; how many stairs to enter and exit house; how many stairs within house?
- Does the patient have the following or need of the following:
  - **Bathroom: (Most likely place to fall!)**
    - *Raised toilet seat, shower chair, grab bars in shower/bath or around bathroom*
  - Kitchen:
    - *Ample, non-spoiled food in fridge and pantry, safe cooking surfaces, fire extinguisher and fire alarms*
  - Bedroom:
    - *Unsecured/loose throw rugs, hard surfaces or corners near bed, need for grab bar on bed*
  - Other:
    - *Evidence of hoarding or severe clutter, pets that could trip them, slip or fall hazards at entrances and exits*

Lewy Body Dementia: Chicken & Egg, Under Umbrella

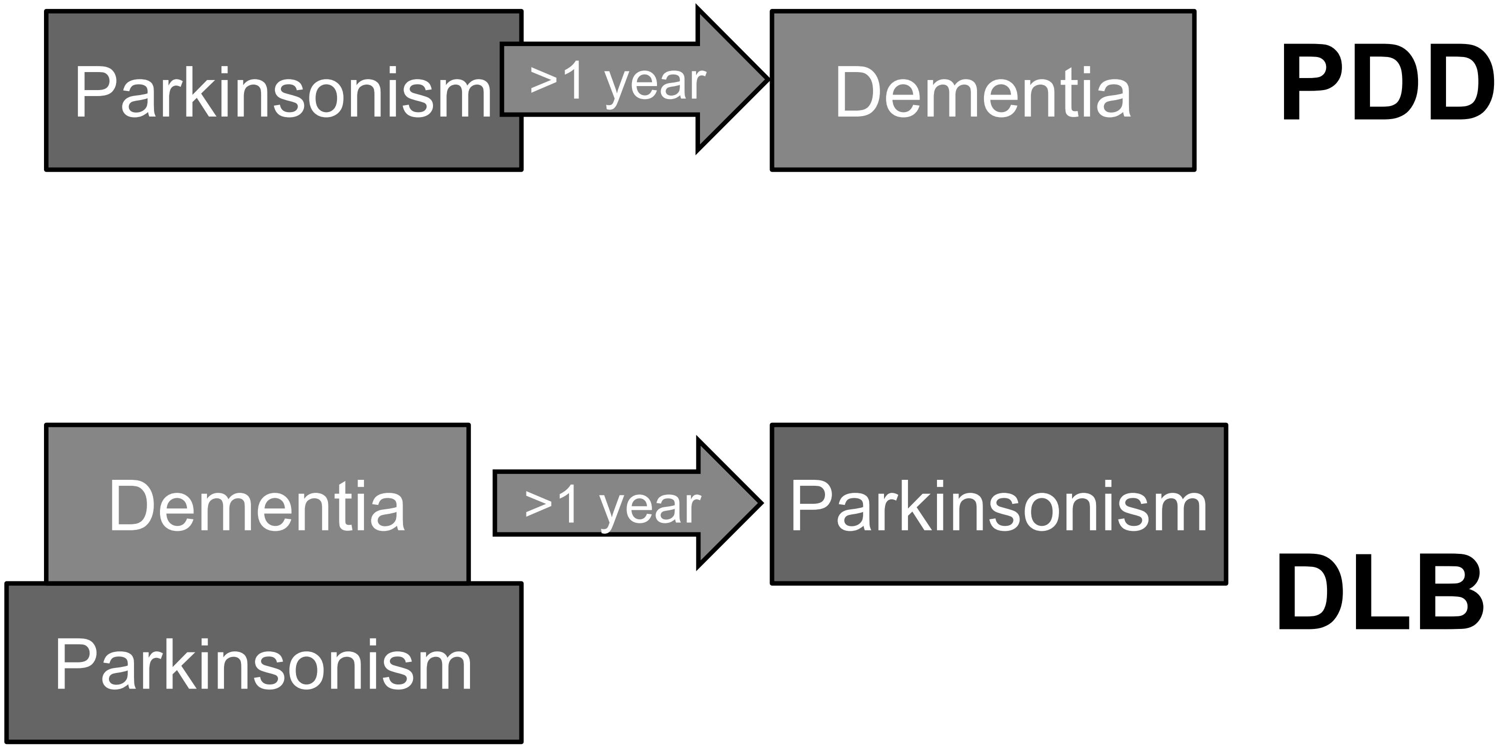

Atypical Parkinsonism

- **DLB:** Cognitive decline and cognitive fluctuations **early** or at the same time as motor changes; hallucinations
- **MSA:** Autonomic dysfunction (orthostatic hypotension, severe urinary dysfunction and/or constipation); parkinsonism or ataxia; early falls
- **PSP:** Falls early, often, and backwards; impaired eye movements and surprised facial expression; dysphagia; symmetric stiffness
- **CBS:** Falls, VERY asymmetric rigidity, alien limb, apraxia

Final surveys

- Navigate to survey link

1. Answer SAME brief anonymous questions that will help us connect pre surveys with post surveys: First initial of first name, color of first car, childhood home street name
2. Interpersonal reactivity index
3. Two brief PD knowledge tests

- Once you're done, you will automatically be redirected to the Rush Continuing Education website to claim your CE credits

Link provided during presentation

THANK YOU!

Parkinson's Foundation
