# Supplementary material for: The impact and feasibility of a brief, virtual, educational intervention for home healthcare professionals on Parkinson’s Disease and Related Disorders: pilot study of I SEE PD Home
Source: BMC Med Educ. 2022 Jun 28;22:506. doi: 10.1186/s12909-022-03430-7 (PMC9238152; doi:10.1186/s12909-022-03430-7)
Supplement: Supplementary file 3 — Additional file 3. Appendix C. Facilitator guides. [file 12909_2022_3430_MOESM3_ESM.pdf]

Annotated Facilitator Guide: I SEE PD Home Patient & Caregiver Question and Answer Panel

- Serena Hess, RN:
    - Welcome participants back after lunch break.
    - Explain that we have asked several caregivers and patients to join us, each having had experience with home healthcare and were willing to share with us.
    - Ask each patient/caregiver/dyad to briefly introduce themselves, talk for a few minutes about their diagnosis, PD/PRD journey, and summarize their experiences with home healthcare professionals, including disciplines and duration of home healthcare.
    - Directed to the panelists:
        - Can you speak to what worked, and perhaps what didn't work, during your interactions with home healthcare professionals?
        - What do you wish home healthcare professionals knew *prior* to entering your home?
        - Any other reflections from our patients and caregivers about their home healthcare experiences before we open it up for questions from our participants?
- Questions from home healthcare professional participants entered into chat feature during the panel; each question was then read aloud and directed to either patients/caregivers first, or to the I SEE PD Home team (MD, RN, PT, OT, SLP) as appropriate
    - Did you feel you had sufficient education on Parkinson's when first diagnosed? (Directed to patients/caregivers)
    - What are some cues or different devices that worked best in your home? (Directed to patients/caregivers)
    - It can be difficult to understand PD patients on some days. We've also talked today about cognitive challenges that can impact communication. Do you have suggestions on how to converse effectively with PD patients? (Directed to patients/caregivers; moderator then sought additional comments by I SEE PD Home MD, SLP)

Annotated Facilitator Guide: I SEE PD Home Expert Question and Answer Panel

- Jori Fleisher, MD MSCE:
  - Brief re-introduction of team, including Serena Hess, RN MSN; Lauren Andersen, PT DPT NCS CBIS; Kristie Trenkle, MOT, OTR/L CLT; Pat Brown, MA CCC/SLP
  - Encourage participants to unmute and ask questions aloud, or type questions into chat feature, based on presentations, breakout sessions, or specific questions based on experience.
  - "Lightning round" question posed by Dr. Fleisher to rest of I SEE PD Home Team: Best tip, equipment, or life hack under $10 that you constantly recommend to patients?
  - Questions posed by participants (discipline included, if noted by participant):
    - (Occupational therapist asked): When people are having medication changes, they're reporting different symptoms, we're seeing different things, I've tried in the past to come up with different symptom trackers. So, my question is, is that helpful? And if so, what should we be tracking? Are there good resources for that?
      - Parkinson's symptom diaries discussed at length, with resources and website links recommended.
    - How do you approach a neurologist that diagnoses someone with Parkinson's but this person is not really presenting with Parkinson's symptoms and not responding to meds?
      - MD invited participant to share more to clarify symptoms; based on highly unusual presentation of sudden onset of Parkinson's Disease symptoms immediately following a surgical procedure, along with new, non-physiologic, distractible, waxing and waning sensory and motor symptoms occurring on subsequent days, with extensive and unremarkable workup, the diagnosis of Functional Neurologic Disorder was introduced and discussed, with resources provided.
    - Any phone or any equipment that works well with tremors?
    - Any tips about incontinence products? Especially for nighttime?
    - Tips for encouraging hydration?
    - What kind of urinary dysfunction happens in parkinsonism?
  - Final parting thoughts from each I SEE PD Home expert:
    - Lauren Andersen, PT DPT NCS CBIS: "Keep it moving however you can; you don't need to be fancy. Just tell people to go for a walk is the best thing you can recommend"
    - Serena Hess, RN MSN: "Check blood pressure, check meds, see if they have a UTI, and if they have had a bowel movement"
    - Pat Brown, MA CCC/SLP: "You've got to eat, drink, and talk, and do something every day to maintain those things"
    - Jori Fleisher, MD MSCE: "You have to keep moving if you want to keep moving; the right exercise is the one that you'll do"
